# Metabolic remodeling and cardiac dysfunction in left ventricular noncompaction: Insights from the MYH7 Q315R model

**DOI:** 10.1371/journal.pone.0336131

**Published:** 2025-11-14

**Authors:** Shinya Takarada, Yukiko Hata, Keisuke Yaku, Hironori Izumi, Kazuki Fujii, Masaaki Omura, Ichiro Takasaki, Kaori Tsuboi, Mako Okabe, Nariaki Miyao, Hideyuki Nakaoka, Keijiro Ibuki, Sayaka Ozawa, Takashi Nakagawa, Hideyuki Hasegawa, Shojiro Ichimata, Naoki Nishida, Hisashi Mori, Yuko Yanagibashi, Keizo Takao, Fukiko Ichida, Keiichi Hirono

**Affiliations:** 1 Department of Pediatrics, Faculty of Medicine, University of Toyama, Sugitani, Toyama, Japan; 2 Department of Legal Medicine, Faculty of Medicine, University of Toyama, Sugitani, Toyama, Japan; 3 Department of Molecular and Medical Pharmacology, Faculty of Medicine, University of Toyama, Sugitani, Toyama, Japan; 4 Department of Molecular Neuroscience, Faculty of Medicine, University of Toyama, Sugitani, Toyama, Japan; 5 Department of Behavioral Physiology, Faculty of Medicine, University of Toyama, Sugitani, Toyama, Japan; 6 Life Science Research Center, University of Toyama, Sugitani, Toyama, Japan; 7 Faculty of Engineering, University of Toyama, Toyama, Japan; 8 Laboratory of Pharmacology, Faculty of Science and Engineering, University of Toyama, Toyama, Japan; 9 Department of Pediatrics, International University of Health and Welfare, Tokyo, Japan; Okayama University: Okayama Daigaku, JAPAN

## Abstract

Left ventricular noncompaction (LVNC) is a form of cardiomyopathy characterized by excessive trabeculation and a thin compacted myocardial layer. Variants in *MYH7*, which encodes the β-myosin heavy chain, are among the most commonly identified genetic causes of LVNC. Despite its clinical relevance, the metabolic disturbances associated with LVNC remain poorly understood, and the pathophysiological mechanisms have not been investigated in an animal model of *MYH7*-related LVNC. To address this gap, we generated a mouse model carrying the human *MYH7* Gln315Arg (Q315R) variant, a representative mutation linked to LVNC. Mice with the *MYH7* Q315R variant exhibited key features of LVNC, including impaired diastolic function, reduced contractility, and excessive trabeculations extending across the ventricular walls. Metabolomic analysis revealed significant metabolic remodeling, characterized by suppressed glycolysis, lipid oxidation, and tricarboxylic acid (TCA) cycle activity. Levels of key intermediates, including glucose-6-phosphate, pyruvate, and acetyl-CoA, were reduced, along with downregulated expression of glycolytic and mitochondrial genes. Additionally, alterations in the pentose phosphate pathway indicated impaired nucleic acid synthesis, while an increased lactate-to-pyruvate ratio suggested a metabolic shift toward anaerobic glycolysis. This study underscores the critical role of metabolic inflexibility—marked by suppression of glycolysis, lipid metabolism, and TCA cycle activity—in the pathophysiology of LVNC. Targeting these dysregulated metabolic pathways, particularly by enhancing mitochondrial function and restoring metabolic adaptability, presents a potential therapeutic strategy for LVNC treatment.

## Introduction

Left ventricular noncompaction (LVNC) is a cardiomyopathy characterized by a hypoplastic compacted myocardial layer and a prominent noncompacted trabecular layer. LVNC is frequently familial and is classified as an inherited cardiomyopathy in the 2006 American Heart Association classification of primary cardiomyopathies [1]. Variants in sarcomeric genes, particularly *MYH7*, have been identified as major contributors to LVNC, with *MYH7* variants being among the most commonly detected in pediatric cases [2,3].

In conditions such as heart failure and cardiomyopathy, impaired metabolic flexibility leads to energy deficiency and the progression of cardiac dysfunction [4]. For instance, in dilated cardiomyopathy (DCM), suppression of glycolysis and a metabolic shift toward amino acid utilization have been linked to the inability to meet the increased adenosine triphosphate (ATP) demand of stressed cardiomyocytes [5]. Mitochondrial dysfunction further exacerbates this issue by increasing reactive oxygen species (ROS) production, contributing to cellular damage and disease progression [6]. However, the metabolic alterations associated with LVNC remain largely unexplored, and no studies have yet examined myocardial energy metabolism in an animal model of *MYH7*-associated LVNC.

In this study, we generated a mouse model carrying the *MYH7* Gln315Arg (Q315R) variant, which was identified in a human patient with LVNC. Our aim was to evaluate the effects of this variant on myocardial metabolism and function. We hypothesized that the *MYH7* Q315R variant alters energy substrate utilization, resulting in metabolic remodeling in the myocardium and contributing to the progression of LVNC and heart failure. Through echocardiographic analysis, histological examination, and metabolomic profiling, we sought to identify the metabolic mechanisms underlying LVNC and explore potential therapeutic targets for this condition.

## Materials and methods

This study was conducted in accordance with the ethical guidelines of the 1975 Declaration of Helsinki and was approved in advance by the Research Ethics Committee of the University of Toyama, Japan (approval no: I2014003). Informed consent was obtained from all patients or their parents following institutional guidelines. All animal experiments were approved by the Animal Experiment Committee of the University of Toyama (approval no: A2020UH-2 and A2019OPR-1), and breeding was carried out in compliance with the University of Toyama’s “Guidelines for the Care and Use of Laboratory Animals.”

### Clinical patient data

Clinical data, including age, gender, cardiac disease diagnosis, electrocardiogram, echocardiogram, and cardiac magnetic resonance imaging findings, were collected.

### Genetic testing of patients

Next-generation sequencing (NGS) was conducted to analyze 203 genes associated with cardiac diseases, including cardiomyopathy and channelopathies, using the Ion PGM System (Life Technologies, Carlsbad, CA, USA). Two polymerase chain reaction (PCR) primer pools were used to generate 1,870 amplicons for the custom panel, which was utilized to construct the target amplicon library. DNA samples were prepared using the custom panel and the Ion AmpliSeq Library Kit v2.0 (Life Technologies, Carlsbad, CA, USA) for PCR amplification. Each sample was barcoded with the Ion Xpress Barcode Adapters Kit (Life Technologies) and pooled at equimolar concentrations. Emulsion PCR and Ion Sphere Particle (ISP) enrichment were performed using the Ion PGM Hi-Q OT2 Kit (Life Technologies) following the manufacturer’s instructions. ISPs were then loaded onto 316 chips and sequenced using the Ion PGM Hi-Q Sequencing Kit (Life Technologies).

### Sanger sequencing

Candidate pathogenic variants that met the selection criteria were validated through Sanger sequencing. Bi-directional direct sequencing of the amplified fragments was performed using the BigDye Terminator v3.1 Cycle Sequencing Kit (Applied Biosystems, Foster City, CA) and an ABI 3130xl automated sequencer (Applied Biosystems).

### Data analysis and variant classification

Primary, secondary, and tertiary analyses, including signal processing optimization, base-calling, sequence alignment, and variant analysis, were conducted using Torrent Suite and Ion Reporter Software 5.0 (Life Technologies). Minor allele frequencies were estimated using the Genome Aggregation Database (gnomAD) and the Japanese allele frequency data from the Tohoku University Tohoku Medical Megabank Organization (Japanese Multi-omics Reference Panel [jMorp]; https://jmorp.megabank.tohoku.ac.jp) for variant filtering. High-impact variants (start loss, stop gain, stop loss, frameshift, and splice acceptor/donor) and moderate-impact variants (missense, in-frame insertion/deletion, and protein-altering) were filtered at a frequency threshold of <0.0005 in gnomAD and ToMMo 8.3KJPN, with a CADD score of ≥20.

The pathogenicity of potential variants was evaluated using five *in silico* predictive algorithms: FATHMM, SIFT, Align GVGD, MutationTaster2, and PolyPhen-2. Only variants classified as “pathogenic” by at least four of the five tools were selected.

### Generation of *MYH7* Q315R variant mice

C57BL/6J mice (Japan SLC, Shizuoka, Japan) were used to generate founder mice heterozygous for the *MYH7* Q315R variant (*MYH7* Q315R/+) using the CRISPR-Cas9 system.

The guide RNA (gRNA) sequence 5-GATTATGCGTTCATCTCCCG-3 and single-stranded oligodeoxynucleotide (ssODN) sequence 5-CCAGGGGCTGTGGCACACCCGAACCACATTCCTTCCACTTGTAGACATGCTGCTGATCACCAACAACCCCTACGATTATGCGTTCATCTCCCGAGGAGAGACGACTGTGGCCTCCATTGATGACTCTGAAGAGCTCATGGCCACGGATGTAAGTATGCGAGTTCTCAGCTACGGGCAGACACTTGGTCG-3 were used. The methods of CRISPR/Cas9 followed previously reported procedures [7]. Fertilized eggs were implanted in SLC:ICR mice (Japan SLC, Shizuoka, Japan), which served as surrogate mothers. Founder *MYH7* Q315R variant mice were crossed with C57BL/6J (Japan SLC, Shizuoka, Japan) initially, however, to analyze the *MYH7* Q315R variant in C57BL/6NJcl background, we crossed heterozygote *MYH7* Q315R variant mice (*MYH7* Q315R/+) with C57BL/6NJcl (CLEA Japan, Inc. Tokyo, Japan). After at least three generations, we subjected *MYH7* Q315R variant mice to experiments as C57BL/6NJcl background line. Furthermore, these C57BL/6NJcl background variant mice were backcrossed at least 3 times C57BL/6J to establish C57BL/6J background line.

For genotyping, the forward primer 5’- GAGCCTCTCTCTTGGCCTGTTTGC-3’ and reverse primer 5’- GTACCATGTGCCACGACCAAGTG-3’ were used. PCR was conducted under the following conditions: 94°C for 1 min, 98°C for 10 s, 62°C for 5 s, 68°C for 1 s (35 cycles), and 72°C for 1 min, followed by a hold at 4°C. The *MYH7* gene sequences of the mice were confirmed through sequencing after PCR amplification.

### Animals

All animals were maintained under standard laboratory conditions, including a 12-h light/dark cycle (lights on at 7 am), a temperature of 22°C ± 2°C, and a humidity level of 50% ± 10%. Food and water were provided ad libitum. The mice were housed in a barrier facility and fed a solid diet. Wild-type mice were the littermates of the *MYH7* Q315R variant mice. To eliminate sex bias, wild-type, heterozygote *MYH7* Q315R variant (*MYH7* Q315R/+), and homozygote (*MYH7* Q315R/Q315R) mice were age-matched to 10 weeks.

### Echocardiography

Echocardiographic recordings followed the definitions outlined in *Mouse in Biomedical Research* (2nd Edition, 2007), using 10-week-old mice as young adult models.^18^ Age-matched wild-type mice served as controls. Mice were sedated with 5% isoflurane at an air flow rate of 1 L/min in an induction chamber, followed by 1%–2% isoflurane for maintenance of sedation. Echocardiography was performed using the SONIMAGE HS1 ultrasound system (KONICA MINOLTA) with an ultra-wideband linear probe (L18-4), with mice positioned supine and chest hair carefully shaved. Contractile function and left ventricular diameter were assessed from M-mode short-axis images (including the anterior and posterior medial papillary muscles) at a heart rate of 400–450 beats/min. Measurements included left ventricular end-diastolic diameter (LVEDD), left ventricular end-systolic diameter (LVESD), left ventricular anterior and posterior wall thicknesses at end diastole and end systole, and fractional shortening (FS). To assess left ventricular diastolic capacity, trans-mitral inflow was measured using pulsed-wave Doppler in the apical four-chamber view, with E and A waves recorded. These measurements were performed by at least two personnel, including a pediatric cardiologist, with three heartbeats averaged for each measurement. After echocardiography, the mice were euthanized with an intraperitoneal injection of sodium pentobarbital (100 µg/g body weight [BW]), and the hearts were harvested. Heart weight (HW) was measured after washing off as much blood as possible and wiping off any remaining water. HW was normalized to BW to account for individual variations in body and heart size.

### Histological analysis

The excised hearts were fixed overnight in 4% paraformaldehyde, then cut horizontally at the apex in the short axis of the left ventricle, and embedded in paraffin to prepare sections. To examine histological changes from the apex to the papillary muscle, 0.5-mm sections were made from the cut surface and used as specimens. Tissue specimens were stained with hematoxylin and eosin and Elastica–Masson to assess myocardial fibrosis. The thickness of the non-dense and dense sublayers, as well as the fibrotic tissue area, were measured using ImageJ (US National Institutes of Health, Bethesda, MD).

The thickness of the compacted and noncompacted layers was measured in heart sample where the non-dense sublayers were prominent. Clinically, LVNC is characterized by trabeculations extending from the lateral to the posterior wall. The ventricular wall was divided into six equal regions along the circumference. The thickness of the non-dense subcutaneous layer was measured, with trabeculations, endocardial depression, and papillary muscles excluded from the analysis.

The fibrotic tissue area and total myocardial tissue were measured using the following procedure. Histological images, excluding the right ventricle, were analyzed. First, ImageJ was used to separate the RGB tissue image into three independent channels (red, green, and blue). The fibrotic tissue area was quantified by applying a defined threshold to the blue channel. The entire myocardial tissue area was measured by applying a threshold value to the 8-bit converted image to extract the full myocardial tissue. Each threshold was determined empirically to ensure that the extent of fibrotic and nonfibrotic tissues corresponded with the original tissue sample. The same threshold was applied to all images. (Reference images are shown in S1 Fig).

### Isoproterenol stimulation to create myocardial injury

To examine the impact of a stress environment on the phenotype, isoproterenol stimulation was applied to the mice following a previous study protocol.^19,20^ Isoproterenol hydrochloride, a synthetic nonselective β-adrenergic agonist, was dissolved in physiological saline. A dose of isoproterenol (30 μg/g BW) was administered subcutaneously to each group of mice at 8–9 weeks of age. The injections were given once daily for 7 days. Cardiac function was assessed by echocardiography 4–7 days after the final injection, and the hearts were collected 7 days post-injection.

### RNA expression analysis by microarray and real-time PCR

Total RNA was extracted from tissue samples, including the free wall and posterior wall of the left ventricular apex, in both mutated and wild-type mice, using RNeasy Mini Kits (QIAGEN). The RNA quality and quantity were assessed with an Eppendorf BioPhotometer D30 (Eppendorf), and samples were stored at −80°C for future use. RNA amplification was performed using the GeneChip™ WT PLUS Reagent Kit (Thermo Fisher Scientific) following the manufacturer’s instructions. Fragment processing and labeling were conducted with the GeneChip™ WT Terminal Labeling Kit (Thermo Fisher Scientific), as per the manufacturer’s instructions. Transcriptome analysis was carried out using Clariom™ D Arrays (Thermo Fisher Scientific), and data were analyzed using Transcriptome Analysis Console software (v.4.0. 1.36; Thermo Fisher Scientific) with results expressed as a quantitative log2 metric.

### Real-time reverse transcription (RT)-PCR

For real-time RT-PCR, cDNA synthesis was carried out from 300 ng of RNA using SuperScript Reverse Transcriptase (Invitrogen). The reaction was performed with THUNDERBIRD 1 SYBR qPCR Mix (Toyobo Co., Ltd., Osaka, Japan). All experiments were conducted in triplicate. The expression levels were measured using the Thermal Cycler Dice Real Time System (Takara Bio) and compared using the ΔΔCt method, where the threshold cycle difference between the target gene and *Gapdh* was assessed. The primers used for the real-time RT-PCR experiments are listed in S1 Table.

### Metabolite extraction from tissues

The excised animal tissues were immediately frozen in liquid nitrogen and stored at −80°C until use. The frozen tissues were weighed and immersed in a 50% methanol/50% water solution at 30 mg/600 μL. The tissues were homogenized using a multi-bead shocker (Yasui Kikai, Japan). After centrifugation, the supernatant was collected into a new tube. Next, 600 μL of chloroform was added, and the solution was vortexed for 10 s. The mixture was centrifuged at 13,000 × g for 10 min at 4°C. The upper (aqueous) phase was transferred to a new tube, and the process was repeated. Finally, the aqueous phase was dried and reconstituted in liquid chromatography (LC)/ mass spectrometry (MS) grade water, followed by filtration using a 0.45-μm Millex filter unit (Merck Millipore, USA).

### Metabolite measurements by liquid chromatography (LC)/mass spectrometry (MS) and gas chromatography (GC)/MS

Metabolite measurements by LC/MS were performed using multiple reaction monitoring with an Agilent 6460 Triple Quad mass spectrometer paired with the Agilent 1290 HPLC system, following previously described MS settings and chromatographic conditions.^21^ TCA cycle intermediates and amino acids were analyzed by selective ion monitoring using an Agilent 5977 MSD Single Quad mass spectrometer coupled with an Agilent 7890 gas chromatograph. GC/MS separation was conducted using a 30-cm DB5-MS column and a 10-m DuraGuard pre-column, with helium as the carrier gas at a constant flow rate of 1.1 mL/min. The temperature profile started at 60°C for 1 min, increased to 325°C at a rate of 10°C/min, and was held at 325°C for 10 min. Metabolites were quantified as relative abundance using MassHunter Quantitative Software via area integration. Area measurements were verified by two analysts.

### Statistical analysis

Statistical analysis of BW, HW, echocardiography, and tissue morphology changes was conducted using JMP (version 15; SAS Institute, Cary, NC). Continuous variables are presented as mean ± standard deviation, while categorical variables are shown as number (percentage). A one-way analysis of variance was performed to compare the three groups, and for variables with statistically significant differences, the Tukey–Kramer test was used for pairwise comparisons. The unpaired Student’s t-test was applied for two-group comparisons. A *p*-value of less than 0.05 was considered statistically significant.

## Results

### Patient characteristics

This study screened 206 patients clinically diagnosed with LVNC using NGS technology. In silico analysis identified 87 patients with pathogenic variants, including 25 with *MYH7* gene variants. One patient had a missense variant in *MYH7*, causing a glutamine (Q) to arginine (R) substitution at position 315 (S2 Fig). This patient, a boy with a heart murmur at 1 month, was diagnosed with LVNC at 1 year old. Echocardiography revealed reduced left ventricular contractility and a noncompacted layer predominantly in the left ventricular apex and posterior wall. There was no evidence of congenital heart disease, external malformations, or developmental delay. Genetic testing found no reports of this variant in gnomAD or ToMMo 8.3KJPN databases. Furthermore, several in silico algorithms indicated the variant’s high pathogenic potential (S2 Table).

### Generation of *MYH7* Q315R variant mice crossed with C57BL/6NJcl sub-strain mice

We generated *MYH7* Q315R variant mice using the method described above. *MYH7* Q315R variant mice were obtained from the C57BL/6J strain. These *MYH7* Q315R/ + mice showed mild diastolic dysfunction compared to wild-type mice, but did not exhibit the reduced contractility characteristic of human LVNC, and no noncompaction layer was observed histologically (S3 Fig and S3 Table). Genotypic differences, such as single nucleotide polymorphisms, may contribute to the development of heart failure by the *MYH7* Q315R variant, as similar genetic mutations can cause different phenotypes in various mouse strains.^6^ For this reason, we crossed the *MYH7* Q315R variant mice with the C57BL/6NJcl sub-strain. After three or more generations of crosses, we obtained *MYH7* Q315R/+ and the *MYH7* Q315R/Q315R C57BL/6NJcl mice. The following experimental results were based on C57BL/6NJcl mice.

### Echocardiographic findings in *MYH7* Q315R/Q315R mice revealed reduced cardiac contractility and increased left ventricular volume

Echocardiographic data for the wild-type, *MYH7* Q315R/+, and *MYH7*Q315R/Q315R groups are presented in Table 1. The *MYH7* Q315R/Q315R group showed significantly smaller LVESD and FS and significantly larger LVEDD compared to the wild-type group. These findings indicate impaired contractility in *MYH7* Q315R/Q315R mice, leading to an increased left ventricular volume load. Furthermore, the E/A ratio was significantly lower in the *MYH7* Q315R/Q315R group compared to the other groups, indicating the presence of diastolic dysfunction in these mice.

**Table 1:** Comparative analysis of left ventricular noncompaction and compaction layers across *MYH7* Q315R mouse variants.

	Wild-type	*MYH7* Q315R/+	*MYH7* Q315R/Q315R	*p*-value
BW (g)	25.5 ± 3.29	24.5 ± 3.01	25.4 ± 3.48	0.8421
HR (min)	427.7 ± 34.08	445.2 ± 25.2	430.5 ± 14.7	0.4153
LVEDD (mm)	3.9 ± 0.3	3.7 ± 0.2	4.1 ± 0.36^†^	0.0340
LVESD (mm)	2.47 ± 0.40	2.6 ± 0.30	3.1 ± 0.36^† †^	0.0155
AWD (mm)	0.74 ± 0.16	0.71 ± 0.18	0.66 ± 0.05	0.5282
PWD (mm)	0.76 ± 0.05	0.77 ± 0.17	0.8 ± 0.14	0.8264
FS (%)	36.5 ± 6.6	29.1 ± 5.6	26.0 ± 3.9^† †^	0.0063
E (cm/s)	62.5 ± 6.12	58.5 ± 3.21	51.4 ± 3.56^† †^	0.0088
A (cm/s)	44.9 ± 6.13	45.1 ± 4.68	46.7 ± 1.15	0.7905
E/A (cm/s)	1.48 ± 0.20	1.31 ± 0.15	0.92 ± 0.15^† †^**	0.0049
HW (mg)	122.1 ± 12.6	121.5 ± 10.5	128.5 ± 25.2	0.7095
HW/BW	5.10 ± 0.41	4.97 ± 0.33	5.04 ± 0.49	0.8492

BW, body weight; HR, heart rate; LVEDD, left ventricular end-diastolic diameter; LVESD, left ventricular end-systolic diameter; AWD, left ventricular anterior wall thickness at end diastole; PWD, left ventricular posterior wall thickness at end diastole; FS, fractional shortening; E, trans-mitral early wave; A, trans-mitral atrial wave; and HW, heart weight. Values that were statistically significantly different from wild-type are indicated. Statistically significantly differences between *MYH7* Q315R+/- and *MYH7* Q315R+/+ mice.

### The *MYH7* Q315R variant mice displayed significant trabeculation in the left ventricle

**Fig 1:**
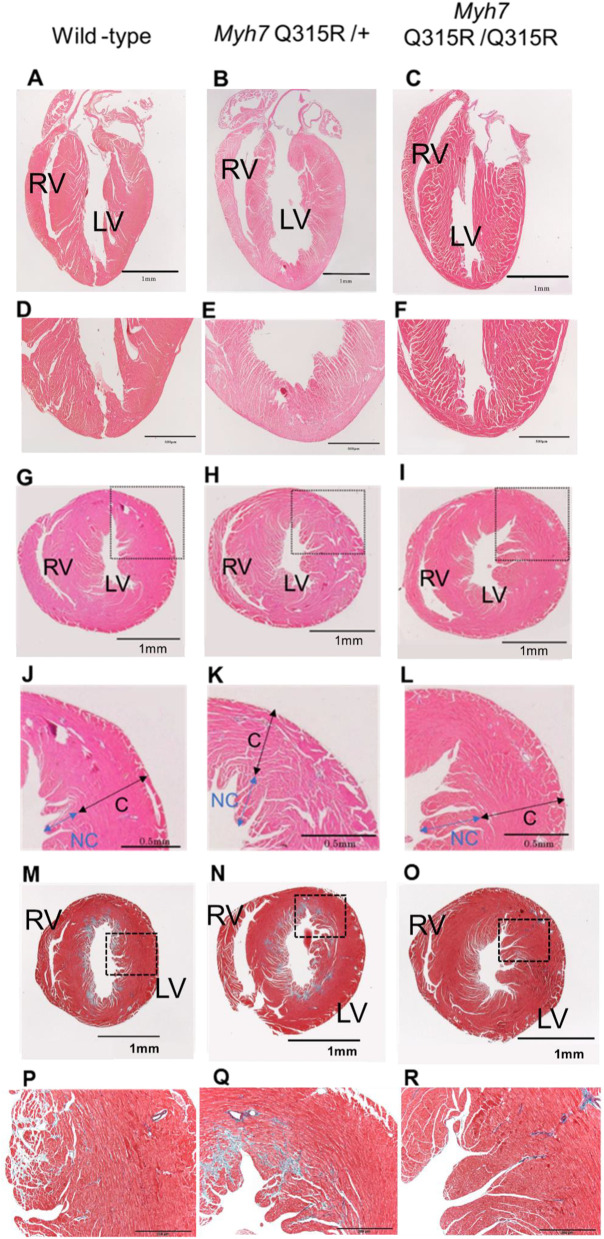
Distinctive left ventricular histology and fibrosis in *MYH7* Q315R mice. Representative hematoxylin-eosin staining of the left ventricle in long-axis images from each mouse group (A–C). Magnified views of the apex in long-axis images (D–F). The wild-type mice exhibit a smooth intima at the apex, while in *MYH7* Q315R/Q315R mice, the compacted layer at the apex is thin, and trabeculation is present (arrow). Representative histological hematoxylin-eosin staining in short-axis images for each mouse group (G–I). Magnified images of the anterolateral wall (dotted border) (J–L). The thicknesses of the noncompacted (NC) and compacted (C) layers are shown in the magnified images. Elastica–Masson stained short-axis sections of the apex for each group (A–C). Magnified images of the anterolateral wall (dotted border) (D–F). No significant difference in fibrosis was observed among the groups. RV, right ventricle. LV, left ventricle.

To evaluate the extent of noncompaction, we compared tissues using short-axis images. Trabeculation was observed at the apex of the heart in the *MYH7* Q315R/Q315R group (Fig 1A–1F). We measured the thickness of the myocardial layer, noncompacted layer (NC layer), the compacted layer (C layer), and the NC/C ratio in six segments for each group (Fig 1G–1L). There was no difference in the total myocardial thickness between the groups. However, the NC/C ratio was significantly higher in the anterior, posterolateral, and posterior walls of the *MYH7* Q315R/ + group and in the anterior, anterolateral, and posterolateral walls of the *MYH7* Q315R/Q315R group compared to the wild-type group (Table 2). Representative histology images are shown in Fig 1D–1L. Fibrosis was grossly assessed in Elastica–Masson-stained tissue sections (Fig 1M–1R). Fibrosis in the *MYH7* Q315R/+ group was similar to that in wild-type mice, while the *MYH7* Q315R/Q315R group showed less fibrosis than both wild-type and *MYH7* Q315R/+ mice.

**Table 2:** Regional variations in myocardial layer thickness and NC/C ratios in *MYH7* Q315R mice.

Segmentations	Measured items	Wild-type	*MYH7* Q315R/+	*MYH7* Q315R/Q315R	*p*-value
Anterior	Wall thickness	0.66 ± 0.10	0.60 ± 0.02	0.55 ± 0.06	0.1195
	C layer	0.52 ± 0.12	0.37 ± 0.03^†^	0.35 ± 0.05^†^	0.0152
	NC layer	0.14 ± 0.03	0.23 ± 0.01	0.20 ± 0.03	0.0049
	NC/C ratio	0.29 ± 0.20	0.61 ± 0.01^†^	0.58 ± 0.12^†^	0.0099
Anterolateral	Wall thickness	0.71 ± 0.15	0.74 ± 0.12	0.76 ± 0.17	0.8623
	C layer	0.53 ± 0.12	0.44 ± 0.10	0.42 ± 0.07	0.2482
	NC layer	0.19 ± 0.06	0.30 ± 0.16	0.34 ± 0.11	0.1142
	NC/C ratio	0.32 ± 0.12	0.72 ± 0.43	0.80 ± 0.17^†^	0.0280
Posterolateral	Wall thickness	0.69 ± 0.17	0.71 ± 0.14	0.76 ± 0.14	0.764
	C layer	0.54 ± 0.11	0.45 ± 0.08	0.49 ± 0.11	0.4428
	NC layer	0.15 ± 0.07	0.25 ± 0.07	0.27 ± 0.03^†^	0.0285
	NC/C ratio	0.27 ± 0.10	0.56 ± 0.09^††^	0.57 ± 0.16^††^	0.0026
Posterior	Wall thickness	0.64 ± 0.16	0.61 ± 0.15	0.63 ± 0.04	0.9326
	C layer	0.51 ± 0.11	0.37 ± 0.07	0.41 ± 0.06	0.0853
	NC layer	0.13 ± 0.07	0.24 ± 0.10	0.21 ± 0.04	0.0865
	NC/C ratio	0.26 ± 0.10	0.65 ± 0.24^††^	0.53 ± 0.16	0.0080
Posteroseptal	Wall thickness	0.61 ± 0.17	0.66 ± 0.08	0.51 ± 0.05	0.248
	C layer	0.44 ± 0.13	0.48 ± 0.09	0.29 ± 0.09	0.0766
	NC layer	0.17 ± 0.06	0.18 ± 0.04	0.22 ± 0.06	0.5669
	NC/C ratio	0.39 ± 0.12	0.40 ± 0.22	0.85 ± 0.52	0.0822
Anteroseptal	Wall thickness	0.66 ± 0.16	0.64 ± 0.14	0.58 ± 0.16	0.7110
	C layer	0.45 ± 0.11	0.49 ± 0.12	0.36 ± 0.36	0.3729
	NC layer	0.18 ± 0.75	0.15 ± 0.09	0.21 ± 0.08	0.5914
	NC/C ratio	0.39 ± 0.17	0.33 ± 0.21	0.73 ± 0.41	0.1077

C layer, compacted layer; NC layer, noncompacted layer; NC/C ratio, ratio of the thickness of the NC layer divided by the thickness of the C layer. Statistically significantly differences compared to wild-type values are indicated.

### Isoproterenol exposure exacerbated the cardiac phenotype in the *MYH7* Q315R variant mice by reducing cardiac contractility

To assess the impact of adrenergic stress, echocardiography was performed on each group of mice after isoproterenol administration (S4 Table). Both the *MYH7* Q315R/Q315R and *MYH7* Q315R/ + groups exhibited a significant decrease in FS compared to the wild-type group. The E/A ratio was also significantly lower in both the *MYH7* Q315R/+ and *MYH7* Q315R/Q315R groups than in the wild-type group. In contrast, the HW/BW ratio was significantly higher in the *MYH7* Q315R/ + group than in the wild-type group. The NC/C ratio was greater in the anterolateral wall of the *MYH7* Q315R variant groups and in the posterolateral wall of the *MYH7* Q315R/Q315R group compared to the wild-type group (S5 Table). Fibrosis was observed in all groups, but no significant difference was found among them (S6 Table). Representative histological images showing fibrosis in each group are shown in Fig 1P–1R.

### Metabolomic and gene expression analyses showed reduced metabolites and expression of genes associated with glucose metabolism, lipid metabolism, and mitochondrial function in the *MYH7* Q315R variant mice

After removing missing data from LC/MS and GC/MS, metabolite data were obtained for each sample. Significant variations in metabolites were identified by examining group differences. Enrichment analysis was performed using MetaboAnalyst (version 5.0; MetaboAnalyst/home.xhtml) to identify metabolic pathways strongly related to these metabolites. This analysis revealed pathways related to glucose metabolism, the Warburg effect, glycogenesis, glycolysis, the pentose phosphate pathway (PPP), the urea cycle, ammonia recycling, and amino acid metabolism (e.g., glutamine, arginine, alanine, glycine, serine, and purine metabolism).

**Fig 2:**
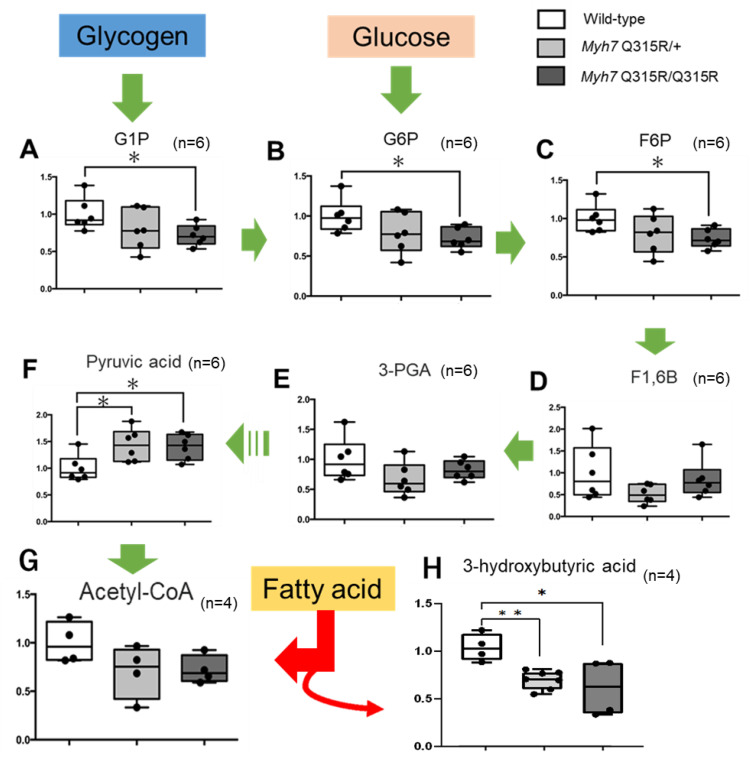
Comparison of glycolytic metabolite concentrations and ketone body concentrations in the *MYH7* Q315R and wild-type mice. Relative levels of glycolysis metabolites and ketone bodies in the hearts of mice were measured by liquid chromatography (LC)/mass spectrometry (MS). The scaled intensities of glucose 1-phosphate (A), glucose 6-phosphate (B), fructose 6-phosphate (C), fructose 1,6-bisphosphate (D), glycerate 3-phosphate (E), pyruvic acid (F), acetyl-CoA (G), and 3-hydroxybutyric acid (H) were determined in cell lysates by LC/MS. Arrows indicate the main flux of glycolysis. Elevated ketone bodies (3-hydroxybutyric acid) reflect acetyl-CoA production from fatty acids (red arrows). G1P, glucose 1-phosphate; G6P, glucose 6-phosphate; F6P, fructose 6-phosphate; F1,6B, fructose 1,6-bisphosphate; 3-PGA, glycerate 3-phosphate; acetyl-CoA, acetyl-coenzyme A. Box-and-whisker plots show relative fold changes compared to wild-type. n is the same for each group and is shown in each graph. * p < 0.05, ** p < 0.01, by unpaired Student’s t-test.

Fig 2 illustrates the glycolysis pathway and a graph showing the measured metabolites in each group. Glucose 1-phosphate, glucose 6-phosphate, and fructose 6-phosphate were notably lower in the *MYH7* Q315R/Q315R group compared to the wild-type group. However, there was no significant difference in blood glucose levels between the wild-type and *MYH7* Q315R/Q315R mice (158.8 ± 35.9 mg/dL vs 151.8 ± 25.1 mg/dL, respectively, *p* = 0.7415). In contrast, pyruvic acid, the end product of glycolysis, was elevated in both the *MYH7* Q315R/Q315R and *MYH7* Q315R/ + groups compared to the wild-type group. Moreover, 3-hydroxybutyric acid (a ketone) levels did not increase in either the *MYH7* Q315R/+ or *MYH7* Q315R/Q315R groups (Fig 2H) and instead showed a decreasing trend. These findings suggest that fatty acid metabolism may not be activated in the *MYH7* Q315R variant mice.

**Fig 3:**
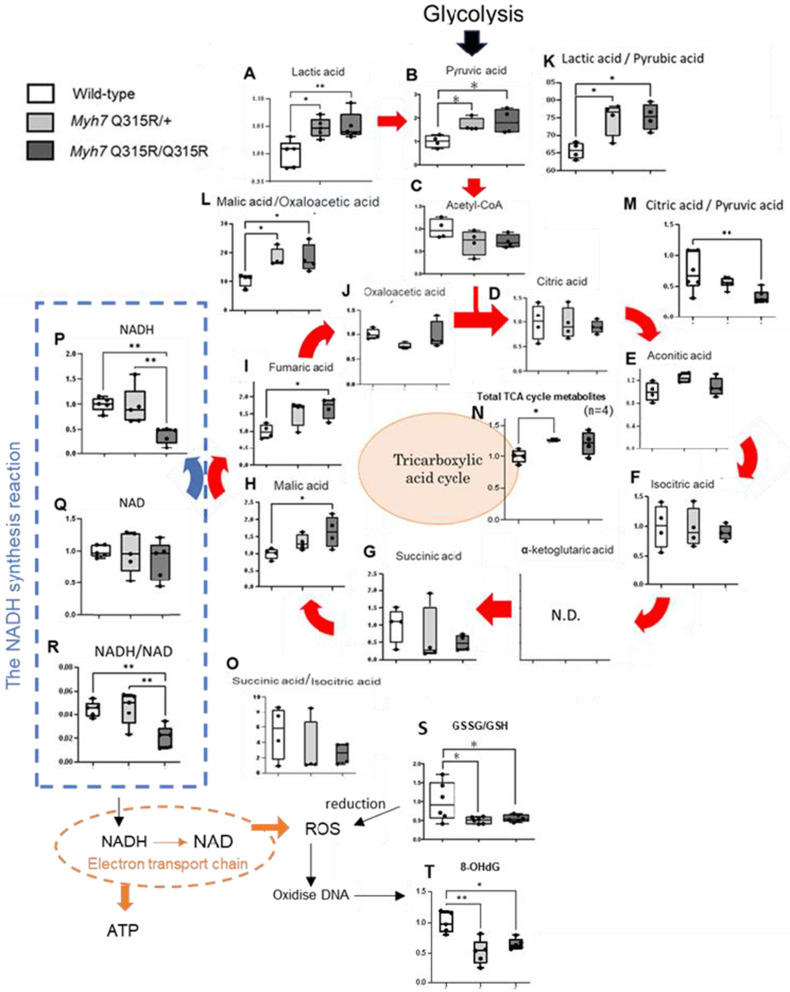
Mitochondrial metabolite profiles in the *MYH7* Q315R variant mice: TCA cycle intermediates, NADH, and oxidative stress markers. The scaled intensities of lactic acid (A), pyruvic acid (B), acetyl-coenzyme A (C), citric acid (D), aconitic acid (E), isocitric acid (F), succinic acid (G), malic acid (H), fumaric acid (I), oxaloacetic acid (J), NADH (P), NAD (Q), GSSG/GSH (S), and 8-OHdG (T) were measured in cell lysates using LC/MS and GC/MS. Ratios of lactic acid/pyruvic acid (K), malic acid/oxaloacetic acid (L), citric acid/pyruvic acid (M), succinic acid/isocitric (O), and NADH/NAD (R) represent the flux between individual metabolites. Total TCA cycle metabolites (N) represent the sum of metabolites (D–J) in each mouse. Red arrows indicate the main flux in the tricarboxylic acid cycle. Pathways and fluxes are as follows: blue, NADH synthesis; orange, electron transfer chain. Acetyl-CoA, acetyl-coenzyme A. NADH, nicotinamide adenine dinucleotide. NAD, oxidized nicotinamide adenine dinucleotide. 8-oHdG, 8-hydroxyguanosine. GSSG, glutathione disulfide. GSH, glutathione. ATP, adenosine triphosphate. ROS, reactive oxygen species. Box-and-whisker plots show relative fold changes compared to the wild-type group. n is consistent across groups and is shown in each graph. * p < 0.05, ** p < 0.01, by unpaired Student’s t-test.

Fig 3 presents the analysis of the TCA cycle and associated metabolites in each group. The oxaloacetate/malate ratio increased in the *MYH7* Q315R variant mice, while the ratio of succinic acid to isocitric acid remained unchanged between the groups (Fig 3L and 3O). A trend toward an increase in the total measurable metabolites of the TCA cycle was observed in the *MYH7* Q315R variant mice (Fig 3N). When intracellular energy demand is low and ATP levels increase, the citrate turnover rate decreases, oxaloacetate is converted back to malate, and the rate-limiting enzyme isocitrate dehydrogenase is inactivated, resulting in reduced isocitrate flux. The above changes in metabolite concentrations may reflect the similar metabolic changes in the TCA cycle. Furthermore, lactate levels were elevated in the *MYH7* Q315R/ + mice, and pyruvate levels were elevated in the *MYH7* Q315R variant mice, along with an increased lactate/pyruvate ratio (Fig 3A, 3B, and 3K). One possible cause of increased lactic acid is a stagnation in the TCA cycle downstream from lactate. Additionally, the citrate/pyruvate ratio showed a decreasing trend in the *MYH7* Q315R/Q315R mice (Fig 3M). Another possible cause of increased lactic acid is enhanced anaerobic metabolism. The lactate/pyruvate ratio increases when glycolysis becomes anaerobic, accompanied by a reduction in the nicotinamide adenine dinucleotide (NADH) produced by glycolysis. Indeed, NADH levels were lower in the *MYH7* variant mice compared to the wild-type mice (Fig 3P). Moreover, the NADH/oxidized nicotinamide adenine dinucleotide (NAD) ratio, an indicator of energy production in the TCA cycle, was lower in the *MYH7* Q315R/Q315R mice than in the wild-type mice (Fig 3Q and 3R). In summary, the *MYH7* Q315R variant mice may exhibit a shift toward anaerobic metabolism, with the TCA cycle potentially suppressed.

8-hydroxyguanosine (8-OHdG) and the ratio of glutathione disulfide to glutathione (GSSG/GSH) are markers of oxidative stress. 8-OHdG is primarily formed when DNA is oxidized by ROS. GSH counteracts ROS-induced oxidative stress by being oxidized to GSSG. Therefore, an increase in ROS production typically leads to a rise in the GSSG/GSH ratio. Fig 3S and 3T show the comparison of 8-OHdG and GSSG/GSH across the groups. The 8-OHdG levels were generally lower in the *MYH7* Q315R/ + group compared to the wild-type group. Similarly, the GSSG/GSH ratio was lower in both the *MYH7* Q315R/+ and *MYH7* Q315R/Q315R groups than in the wild-type group.

Metabolites linked to the PPP and nucleic acid synthesis pathway were lower in *MYH7* Q315R variant mice compared to wild-type mice. The PPP, a branch of the glycolytic pathway, primarily generates metabolites for nucleic acid synthesis. Many intermediate metabolites in the PPP were reduced in both *MYH7* Q315R/+ and *MYH7* Q315R/Q315R mice compared to wild-type mice (S4 Fig). Notably, D-ribose 5-phosphate, which initiates the nucleic acid biosynthetic pathway (S5 Fig), was significantly downregulated in both *MYH7* Q315R/+ and *MYH7* Q315R/Q315R mice. Myo-inositol phosphate (IMP), the end product of nucleic acid synthesis, was also significantly reduced in both *MYH7* Q315R/+ and *MYH7* Q315R/Q315R mice. IMP is converted to adenine and guanine via adenosine monophosphate (AMP) and guanosine monophosphate, respectively. While AMP levels remained similar between the *MYH7* Q315R/+ and wild-type mice, adenine levels were significantly reduced in the *MYH7* Q315R/+ and *MYH7* Q315R/Q315R mice. It is important to note that AMP levels reflect both the nucleic acid synthesis pathway and ATP/adenosine diphosphate metabolism. Overall, steady-state concentrations of metabolites in the PPP and nucleic acid synthesis pathways tended to be lower in the *MYH7* Q315R variant mice.

Several amino acids were altered in the *MYH7* Q315R variant mice compared to the wild-type mice (S6 Fig). However, there were no changes in the expression of enzymes involved in using amino acids as energy substrates, such as transaminases, dehydratases, and amino acid oxidases. It is unclear from this study whether these amino acids were utilized for energy production or altered due to metabolic disorders.

Genes associated with metabolic pathways, which varied according to metabolomic data, were examined through microarray analysis (S8 Table). For metabolism-related genes, thresholds were set at fold change >1.2 or fold change <−1.2 and p < 0.05, compared to the wild-type group. The effects on each gene and metabolic pathway are detailed in S8 Fig.

The expression of *Pfk*, *Pkm1*, and *Pdp2*, genes related to glycolysis, was reduced in *MYH7* Q315R/Q315R mice. *Pfk* and *Pkm1* encode phosphofructokinase and pyruvate kinase, respectively, which are rate-limiting enzymes in glycolysis. The reduced expression of these enzymes suggests that glycolysis flux is suppressed. *Pdp2* encodes pyruvate dehydrogenase phosphatase, which activates pyruvate dehydrogenase and enhances the conversion of pyruvic acid to acetyl-CoA. Therefore, the reduced expression of *Pdp2* supports a suppression of glycolysis.

**Fig 4:**
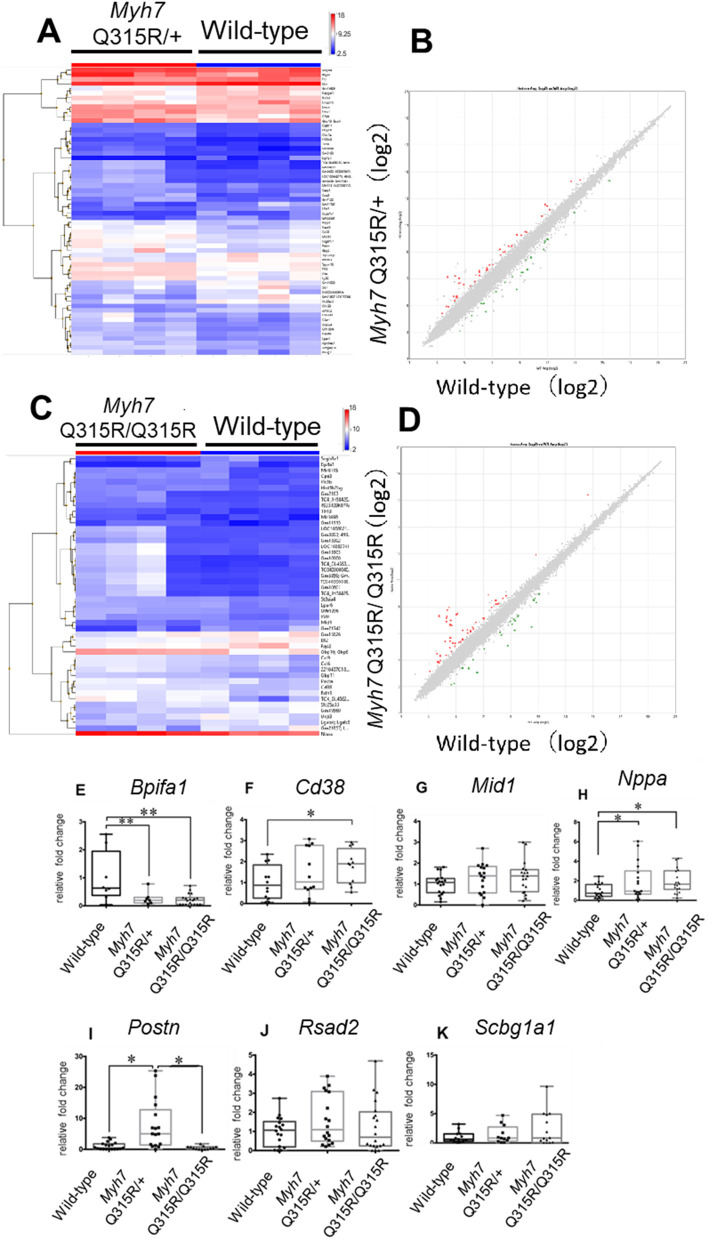
Differential mRNA expression of key metabolic and stress response genes in the *MYH7* Q315R mice. Candidate genes with significantly different mRNA expression between wild-type and transgenic mice, identified by microarray, were validated using real-time polymerase chain reaction (PCR). Clustering maps show significant changes in RNA expression (p < 0.05) greater than twofold or less than −2.0-fold (A and C). Scatter plots depict RNA changes, with red dots indicating increased expression and blue dots indicating decreased expression (B and D). Real-time PCR data show relative gene expression in the hearts of mice from each group, with the average expression of the wild-type group set to 1 (E-K). Box-and-whisker plots display mRNA expression levels for each gene across three or more experiments, normalized by the corresponding glyceraldehyde 3-phosphate dehydrogenase expression. n = 4 per group. * p < 0.05, ** p < 0.01, by unpaired Student’s t-test.

Genes related to lipid metabolism, such as *Slc27a1*, which encodes long-chain fatty acid transport protein 1 (FATP1) that facilitates fatty acid entry into cells, and *Cpt1a*, which transports acyl-CoA into the mitochondria, are both downregulated in *MYH7* Q315R variant mice. *Bdh1*, responsible for the first step in converting ketone bodies to acetyl-CoA in cells, was significantly elevated in *MYH7* Q315R/Q315R mice. Metabolomics analysis indicated a trend toward reduced 3-hydroxybutyrate in *MYH7* Q315R variant mice, suggesting ketone bodies are being utilized to generate acetyl-CoA to compensate for impaired lipid and glucose metabolism. Furthermore, acetyl-CoA, the final product of fatty acid metabolism, showed a decreasing trend in the *MYH7* Q315R variant mice (Fig 4G). These findings suggest that lipid metabolism-driven energy production is suppressed in the hearts of *MYH7* Q315R variant mice.

In terms of mitochondrial function, altered expression of *Cd38* and *Pgc-1α* was prominent in *MYH7* Q315R/Q315R mice. Cd38 degrades NAD, reducing mitochondrial function and insulin sensitivity. The high expression of *Cd38* in *MYH7* Q315R variant mice limits metabolic flexibility. *Pgc-1α*, activated by various cardiac stresses, enhances mitochondrial function, promotes glucose and fatty acid metabolism, and stimulates ATP production. Consequently, the reduced expression of Pgc-1α in *MYH7* variant mice may hamper cardiomyocyte energy production.

The expression of *Sirt2* (encoding Sirtuin 2) and *Pparγ* (encoding peroxisome proliferator-activated receptor gamma) was elevated in the *MYH7* Q315R/ + mice. *Sirt2* enhances fatty acid oxidation and insulin sensitivity, while *Ppar*γ influences adipose tissue expansion and insulin sensitivity. These changes in gene expression in the *MYH7* Q315R/+ mice may be counteract the metabolic changes observed in the *MYH7* Q315R/Q315R mice by increasing glycolysis and lipid oxidation.

### Cellular stress was increased in the hearts of the *MYH7* Q315R variant mice, as indicated by gene expression profiling

Microarray analysis compared the gene expression levels in the heart tissue of young adult mice from each group (Fig 4 and S7 Fig). A total of 112 genes showed significant changes (fold change >2.0 or fold change <−2.0 and p < 0.05) in the *MYH7* Q315R/+ group and 82 genes in the *MYH7* Q315R/Q315R group. Among these, 48 genes (33 upregulated and 15 downregulated) were coding genes in the *MYH7* Q315R/+ group, and 31 genes (19 upregulated and 12 downregulated) were coding genes in the *MYH7* Q315R+/+ group, after excluding noncoding genes and microRNAs. Functional enrichment analysis identified terms related to immune and inflammatory responses (S7 Table). Seven genes were selected for association with heart disease, including five common genes in the *MYH7* Q315R variant mice group and one from each of the *MYH7*Q315R/+ and *MYH7*Q315R/Q315R groups, and were confirmed by real-time PCR. Among these, *Nppa*, a heart failure marker, was upregulated in both the *MYH7* Q315R/+ and *MYH7* Q315R/Q315R groups compared to the wild-type group. *Cd38*, induces chronic inflammation via activation of inflammatory macrophages, was upregulated in the *MYH7* Q315R/Q315R group. *Postn*, which is upregulated in myocardial remodeling after myocardial ischemia, was increased in the *MYH7* Q315R/ + group. In contrast, Bpfi1a was downregulated in both the *MYH7* Q315R/+ and *MYH7* Q315R/Q315R groups compared to the wild-type group.

## Discussion

This is, to the best of our knowledge, the first report demonstrating changes in myocardial energy metabolism in an animal model of LVNC caused by *MYH7* variants. These alterations include reductions in metabolites associated with glycolysis, lipid metabolism, the citric acid cycle, and the PPP. These findings suggest that *MYH7* variants may induce metabolic changes, such as a shift to anaerobic glycolysis and reduced mitochondrial activity, via primary or secondary metabolic remodeling. Moreover, metabolic inflexibility may be crucial in the pathophysiology of LVNC and contribute to its progression.

### Alterations in energy metabolism and their impact on LVNC pathophysiology

The myocardium primarily uses glucose and lipids as energy substrates, and the relative contributions of these substrates change in response to myocardial stress and substrate availability [8]. During stress, when there is an increased demand for ATP, glucose metabolism is usually prioritized because of its efficiency [9]. However, in the *MYH7* Q315R variant mice, both lipid and glucose metabolism are significantly impaired, and TCA cycle activity is reduced. The concurrent impairment of fatty acid metabolism, glycolysis, and mitochondrial function is characteristic of advanced heart failure, a process known as metabolic remodeling. The metabolic changes observed in this study may alight with the pattern of metabolic remodeling seen in heart failure and cardiomyopathy. From this point of view, the metabolic changes induced by the *MYH7* Q315R variants could limit energy supply by impairing myocardial metabolic flexibility. For instance, impaired glucose and lipid metabolism or the TCA cycle can make the myocardium vulnerable to energy deficiency. It is unable to meet the demand for ATP under stress, leading to the progression of cardiac dysfunction and remodeling. Therefore, the metabolic changes observed in this study are considered to provide important insights into the pathophysiology of LVNC and heart failure progression.

Cardiac contractility is impaired in mice with the *MYH7* Q315R variant, and these metabolic findings are similar to those of known metabolic changes in heart failure. However, the absence of increased oxidative stress is noteworthy. Excessive production of ROS, generated in an attempt to sustain ATP production in stressed myocardium with limited substrate switching capacity, may play a significant role in heart failure progression. Metabolic remodeling in heart failure typically involves a decline in energy metabolism, starting with reduced fatty acid metabolism, followed by impaired glucose metabolism and, eventually, mitochondrial dysfunction, leading to a substantial energy deficit in the myocardium [10]. Normally, mitochondrial dysfunction and electron transport chain disruption results in increased NADH accumulation and elevated ROS levels [11]. However, in the *MYH7* Q315R variant mice, NADH levels were lower compared to wild-type mice, and markers of oxidative stress, such as 8-OHdG and GSSG/GSH, were also reduced. Changes in NADH and ROS markers suggest that mitochondrial function in the myocardium of *MYH7* Q315R variant mice is suppressed rather than fully dysfunctional. These findings do not support complete mitochondrial dysfunction but suggest that the metabolic changes in the *MYH7* Q315R variant mice reflect an ongoing cardiac remodeling process that has not yet led to severe mitochondrial failure. Therefore, the lack of significant oxidative stress may help slow the progression of heart failure, suggesting that specific metabolic changes contribute to the pathophysiological progression of LVNC.

### The role of metabolic remodeling in cardiomyopathies

At rest, the heart primarily uses fatty acid metabolism for energy. In heart failure with reduced ejection fraction (HFrEF), increased utilization of alternative metabolic pathways, particularly glycolysis, helps compensate for energy deficits [12]. In contrast, in heart failure with preserved ejection fraction (HFpEF), although TCA cycle metabolites are lower than in HFrEF, fatty acid metabolism is further reduced, and substrate metabolism becomes less flexible [13]. In DCM, early metabolic changes involve suppressed glucose metabolism and reduced TCA cycle intermediates, leading to lower energy production [14]. There are also reductions in metabolites from the PPP and urea cycle in DCM myocardium [15]. In hypertrophic cardiomyopathy (HCM), ATP demand fluctuates in the early stages due to changes in the myocardium’s workload [16]. Over time, as hypoxia develops in the hypertrophied myocardium, anaerobic glycolysis becomes the dominant energy pathway, with most glucose converted to lactate [17]. The metabolic changes in the *MYH7* Q315R variant mice, including decreased concentrations of intermediates in glycolysis and the PPP, resemble those observed in DCM. The suppression of the PPP in *MYH7* Q315R variant mice, alongside the lack of significant myocardial hypertrophy, is consistent with both echocardiographic and histological findings. Although fatty acid metabolism is generally preserved in DCM, the *MYH7* Q315R variant mice fatty acid metabolism may be suppressed. This may be due to differences associated with diastolic dysfunction, which is characteristic of HFpEF [13]. The worsening of cardiac remodeling and progression to heart failure triggered by isoproterenol stress supports the idea that the *MYH7* Q315R variant mice exhibit a phenotype typical of HFpEF. In summary, the results of this study indicate that while LVNC exhibits a metabolic profile more similar to HFpEF and DCM. The observed lack of metabolic flexibility may render the myocardium more vulnerable and increase susceptibility to worsening heart failure under stress conditions such as those induced by ISP loading.

### Therapeutic implications and the role of *MYH7* variants

The *MYH7* gene, located on chromosome 14p12, consists of 41 exons and encodes the β-myosin heavy chain, which is mainly expressed in cardiac muscle. Variants in *MYH7* are a primary cause of LVNC [6]. However, the exact mechanisms through which sarcomeric gene variants, including *MYH7*, contribute to the development of LVNC remain unclear. Recent studies have pointed out that specific amino acid positions within the β-myosin heavy chain are particularly prone to variants, forming hotspots where these variants are more common and tend to cluster in critical functional regions [3]. Notably, over half of the *MYH7* variants associated with LVNC, such as the Q315R variant, are located in the segment-1 region.

Understanding the direct link between *MYH7* variants and cardiac metabolism is crucial for understanding the pathophysiology of LVNC. These variants have also been associated with HCM and DCM, where they influence ATP utilization and β-myosin heavy chain activity, resulting in metabolic changes in the myocardium [18]. Such metabolic alterations may play a significant role in the progression of heart failure in DCM, as the lack of substrate flexibility impairs the ability to maintain adequate ATP production to meet the increased ATP demand of a stressed myocardium [19]. Cardiac dysfunction in DCM is exacerbated by mitochondrial dysfunction, cell death, and abnormal calcium homeostasis in cardiomyocytes [4]. Variants in *MYH7*, which encodes the β-myosin heavy chain, directly affect sarcomeric function and cellular metabolism, as seen in HCM [20]. These variants lead to abnormal myosin–actin interactions, increasing energy consumption and reducing metabolic efficiency [21]. The enhanced contractility associated with *MYH7* variants raises ATP demand, while mitochondrial function—an essential energy source—is impaired, creating a mismatch between energy supply and demand. Specifically, *MYH7* variants disrupt the balance between the super-relaxed (SRX) state and disordered-relaxed (DRX) state of myosin [16,22]. This conformational change increases ATPase activity, raising energy consumption even at rest and placing cardiomyocytes under metabolic stress. The changes in sarcomeric function observed in *MYH7*-related HCM have direct consequences for cardiac metabolism [16,22]. The mutated myosin’s inefficiency in utilizing ATP leads to ATP depletion, prompting compensatory increases in glycolysis and lipid metabolism. However, as these pathways become overwhelmed, reductions in key metabolites like acylcarnitines and free fatty acids, along with decreased oxidative phosphorylation capacity, are observed [23]. This metabolic shift toward anaerobic glycolysis worsens the energy deficit in *MYH7*-variant cardiomyocytes, contributing to impaired contractile function under stress. Additionally, mitochondrial dysfunction in *MYH7*-variant hearts is associated with increased oxidative stress and reduced ATP production. Studies suggest that *MYH7* variants cause structural changes in mitochondria, such as reduced cristae density, further impairing oxidative phosphorylation capacity [16,22].

In summary, the *MYH7* variant disrupts sarcomeric function and metabolic flexibility, creating a harmful cycle of energy depletion, mitochondrial dysfunction, and metabolic stress that accelerates disease progression. Interventions focused on enhancing mitochondrial function and restoring metabolic flexibility may help slow this progression. For example, mitochonic acid-5, currently in clinical trials for mitochondrial diseases, has shown potential in improving mitochondrial ATP production, suggesting it could be a promising therapeutic approach for cardiomyopathy [24]. Additionally, mavacamten, a myosin inhibitor targeting *MYH7*, has been approved for treating HCM by reducing myosin–actin cross-bridge formation and ATP consumption [25]. Omecamtiv mecarbil, which enhances myosin–actin interactions, has proven effective in increasing cardiac contractility, making it a potential treatment for HFrEF [26]. Despite these advances, no therapies currently address the mitochondrial dysfunction directly caused by *MYH7* variants. Therefore, developing treatments that specifically target mitochondrial impairments linked to *MYH7* variants offers a promising direction for heart failure management. Our study provides key insights into the pathophysiological mechanisms linking *MYH7* variants to pathological metabolic changes, highlighting the importance of this therapeutic strategy.

### Study limitations

The gene expression of specific cell types, particularly cardiomyocytes, was not assessed individually using single-cell RNA sequencing. Future studies examining changes in myosin activity in isolated cardiomyocytes, along with proteomic analysis of protein expression, may help to identify the pathways driving the metabolic changes. It is unclear whether the metabolic changes observed in this study are the primary effect a primary or secondary effect of the *MYH7* Q315R variant. Additional experiments with rescue or knock-in models are required to clarify this. The current metabolite measurements only provide a static assessment. Evaluating metabolic pathway flux and activity requires isotope labeling and time-course analysis. The effects of environmental factors, such as age, high-fat diet, and starvation, on myocardial morphology and phenotype were not explored. The intracellular functional evaluation, including ATP levels, fatty acid content, ROS production and mitochondrial function, was not measured directly. These functional assays will be required in future validation studies. We acknowledge the limitations of statistical power. However, we were able to present results with a large effect size and high biological validity. Validation in larger cohorts is necessary.

Additionally, the *MYH7* Q315R/+ and *MYH7* Q315R/Q315R C57BL/6NJcl mice showed reduced cardiac contractility, including diastolic and systolic dysfunction, compared to their littermates, while the *MYH7* Q315R/+ C57BL/6J mice showed only diastolic dysfunction. These differences may be attributed to variations in the localization and timing of *MYH7* gene expression in the mouse heart [27]. Specifically, the introduction of the genetic variant seen in LVNC patients in mice has not resulted in the same noncompacted layer phenotype observed in humans [28]. It is likely that the expression of the *MYH7* gene differs between humans and mice during heart development. Additionally, reported differences in cardiac morphology and function between the C57BL/6J and C57BL/6NJcl mouse strains may contribute [5]. However, the phenotypic differences observed compared to their wild-type siblings suggest that genetic mutations, rather than strain-specific variations, strongly influence the observed phenotype.

## Conclusion

Mice carrying a human *MYH7* gene variant exhibited phenotypes typical of LVNC, including diastolic dysfunction and reduced cardiac contractility. Furthermore, metabolomic and microarray analyses suggest a suppression of key metabolic pathways, such as glycolysis, lipid metabolism, and the TCA cycle, in the *MYH7* Q315R variant mice. The identified changes in substrate metabolites provide important insights into the pathogenesis of LVNC. A deeper understanding of the metabolic mechanisms involved in LVNC may lead to the development of novel therapeutic strategies targeting these pathways.

## Supporting information

S1 Fig Quantification of fibrotic tissue area in the myocardium. Original Elastica–Masson staining (excluding the right ventricle) (A). The total area of myocardial tissue is outlined in black and measured(B). The fibrotic tissue, stained blue, is outlined in black and measured as the fibrosis area (C).
(DOCX)

S2 Fig Genetic identification and validation of the *MYH7* Q315R variant in an LVNC patient. (A) Pedigree of the patient. There is no family history of sudden death or cardiac disease in the parents. (B) DNA sequencing of the patient revealed a single-base missense mutation resulting in the substitution of glutamine (Q) with arginine (R) (black arrow).
(DOCX)

S3 Fig Comparison of cardiac histology between wild-type and *MYH7* Q315R/+ C57BL/6J strain mice. Typical histological hematoxylin-eosin (HE) staining in short-axis images of both groups (A, B). Typical histological Elastica–Masson (EM) staining in short-axis images of both groups (C, D). No significant histological differences were observed between the two groups. RV, right ventricle. LV, left ventricle.
(DOCX)

S4 Fig Suppression of pentose phosphate pathway metabolites in *MYH7* Q315R mouse hearts. The relative levels of pentose phosphate pathway metabolites in mouse hearts were measured by LC/MS. The scaled intensities of glucose 6-phosphate (A), 6-phosphogluconate (B), ribulose 5-phosphate (C), D-xylulose 5-phosphate (D), ribose 5-phosphate (E), glyceraldehyde 3-phosphate (F), sedoheptulose 7-phosphate (G), fructose 6-phosphate (H), and erythrose 4-phosphate (I) were determined in cell lysates. The main flow of the pentose phosphate pathway is indicated by arrows. G6P, glucose-6-phosphate; 6PG, 6-phosphogluconate; Ru5P, ribulose 5-phosphate; Xu5P, D-xylulose 5-phosphate; R5P, ribose 5-phosphate; GAP, glyceraldehyde 3-phosphate; S7P, sedoheptulose 7-phosphate; F6P, fructose 6-phosphate; E4P, erythrose 4-phosphate. The Box-and-whisker diagrams show the relative fold change compared to the wild-type group. n = 6 per group. * p < 0.05, ** p < 0.01, by unpaired Student’s t-test.
(DOCX)

S5 Fig Impaired nucleic acid synthesis and salvage pathways in *MYH7* Q315R mice. The relative levels of nucleic acid synthesis pathway metabolites and salvage pathway metabolites in mouse hearts were measured by LC/MS. The scaled intensities of ribose 5-phosphate (A), phosphoribosyl diphosphate (B), myo-inositol phosphate (C), adenosine monophosphate (D), and adenine (E) were determined in cell lysates. The main flow of the nucleic acid synthesis pathway is indicated by blue arrows. R5P, ribose 5-phosphate; PRPP, phosphoribosyl diphosphate; IMP, myo-inositol phosphate; AMP, adenosine monophosphate; GMP, guanosine monophosphate. Box-and-whisker diagrams show the relative fold change compared to the wild-type group. n = 6 per group. * p < 0.05, ** p < 0.01, by unpaired Student’s t-test.
(DOCX)

S6 Fig Altered amino acid profiles in *MYH7* Q315R mice: implications for energy metabolism. Amino acids that differ between *MYH7* Q315R variant and wild-type mice include glycine (A), serine (B), tryptophan (C), glutamic acid (D), and aspartic acid (E). The box-and-whisker plots show the relative fold change compared to the wild-type group. The sample size (n) is the same for each group and is indicated in each graph. * p < 0.05, ** p < 0.01, by unpaired Student’s t-test.
(DOCX)

S7 Fig Metabolic pathways and genes altered in *MYH7* Q315R variant mice. Heat maps clustering the metabolites in each group of mice. A: Heat map clustering the metabolites measured by LC/MS (n = 6 per group). B: Heat map clustering the metabolites measured by GC/MS (n = 4 per group).
(DOCX)

S8 Fig Changes in expression levels were observed for genes related to glycolysis, fatty acid oxidation and mitochondrial function in the *MYH7* variant mice. *Sirt2* (A) and *Pparγ* (B) activate glycolysis and fatty acid oxidation, which were elevated in *MYH7* Q315R/ + mice. *Pfk* (C), *Pkm-1* (E), *Pdp2* (G), and *Bdh1*(F) enhance reactions in their respective pathways. *Slc27a1* (D) encodes a transport protein that facilitates fatty acid uptake into the cytoplasm. *Cpt1a* (H) encodes a transferase that transports acetyl-CoA into the mitochondria. *Pcg-1α* (I) promotes glycolysis, fatty acid oxidation, and mitochondrial function. *Cd38* (J) inhibits glycolysis and mitochondrial function. G6P, glucose 6-phosphate; F6P, fructose 6-phosphate; PEP, phosphoenolpyruvic acid; acetyl-CoA, acetyl-coenzyme A; TCA, tricarboxylic acid cycle; FAO, fatty acid oxidation. A–J are graphs showing fold change of each gene in *MYH7* Q315R variant mice against the wild type mice. n = 4 for each group. * Fold change >1.2 or <−1.2 and p < 0.05, by unpaired Student’s t-test.
(DOCX)

S1 Table Primers used in real-time RT-PCR experiments. (DOCX)

S2 Table Pathogenicity prediction of the *MYH7* Q315R variant. (DOCX)

S3 Table Baseline echocardiographic parameters in wild-type and *MYH7* Q315R/+ C57BL/6J strain mice. BW, body weight; HR, heart rate; LVEDD, left ventricular end-diastolic diameter; LVEDs, left ventricular end-systolic diameter; AWD, left ventricular anterior wall thickness at end diastole; PWD, left ventricular posterior wall thickness at end diastole; FS, fractional shortening; E, trans-mitral early wave; A, trans-mitral atrial wave; and HW, heart weight. Values significantly different from wild-type are indicated.
(DOCX)

S4 Table Echocardiographic data and heart weight of young adult wild-type, *MYH7* Q315R/+, and *MYH7* Q315R/Q315R mice with isoproterenol loading. BW, body weight; HR, heart rate; LVEDD, left ventricular end-diastolic diameter; LVEDs, left ventricular end-systolic diameter; AWD, left ventricular anterior wall thickness at end diastole; PWD, left ventricular posterior wall thickness at end diastole; FS, fractional shortening; E, trans-mitral early wave; A, trans-mitral atrial wave; and HW, heart weight. Values significantly different from wild-type and between *MYH7* Q315R/+ and *MYH7* Q315R/Q315R mice are indicated.
(DOCX)

S5 Table Segmental variations in NC/C ratios across the left ventricle in *MYH7* Q315R mice. C layer, compacted layer; NC layer, noncompacted layer; NC/C ratio, ratio of the thickness of the NC layer divided by the thickness of the C layer. Values significantly different from wild-type are indicated.
(DOCX)

S6 Table Quantitative analysis of myocardial fibrosis in *MYH7* Q315R mouse groups with isoproterenol loading. The fibrotic tissue area (%) is calculated by dividing fibrotic tissue area(mm^2^) by the total area (mm^2^) for each group. No significant differences were observed between the three group.
(DOCX)

S7 Table Functional enrichment analysis of differentially expressed genes in *MYH7* Q315R mice. The table above shows the results for the *MYH7* Q315R/+ group. The table below shows the results for the *MYH7* Q315R/ Q315R group.
(DOCX)

S8 Table Alterations in *MYH7* Q315R variant mice genes associated with metabolism, as revealed by microarray analysis. Fold change and *p*-value were compared with wild-type mice. n = 4 per group. *p*-value was determined by unpaired Student’s t-test.
(DOCX)
