# Supplementary figures and images for: Metabolic remodeling and cardiac dysfunction in left ventricular noncompaction: Insights from the MYH7 Q315R model
Source: PLoS One. 2025 Nov 14;20(11):e0336131. doi: 10.1371/journal.pone.0336131 (PMC12617873; doi:10.1371/journal.pone.0336131)

**S1 Fig. Quantification of fibrotic tissue area in the myocardium**

**
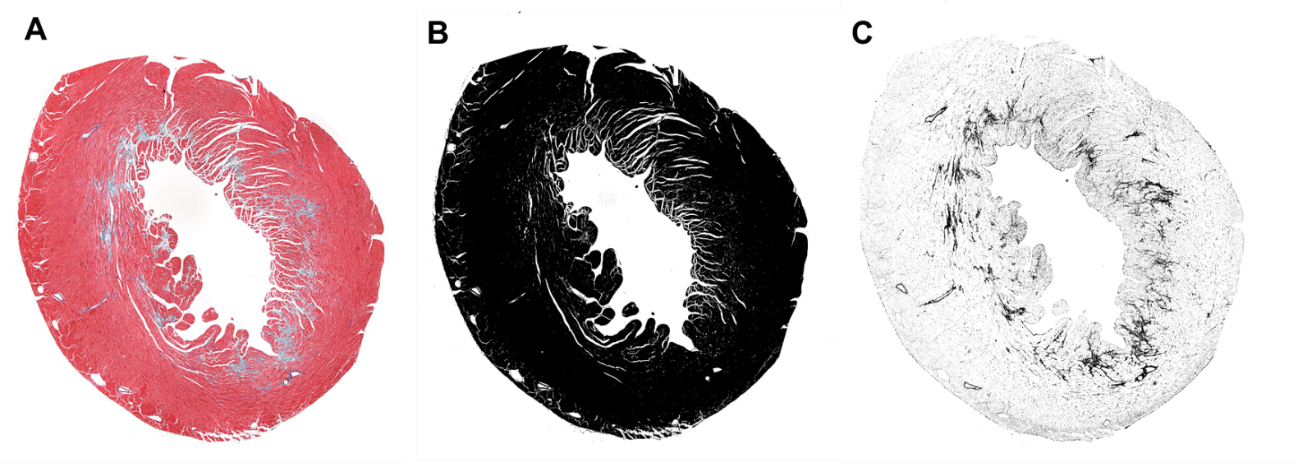
**

Supplement: S1 Fig — Original Elastica–Masson staining (excluding the right ventricle) (A). The total area of myocardial tissue is outlined in black and measured(B). The fibrotic tissue, stained blue, is outlined in black and measured as the fibrosis area (C). (DOCX) [file pone.0336131.s001.docx]

**S2 Fig. Genetic identification and validation of the *MYH7* Q315R variant in an LVNC patient**


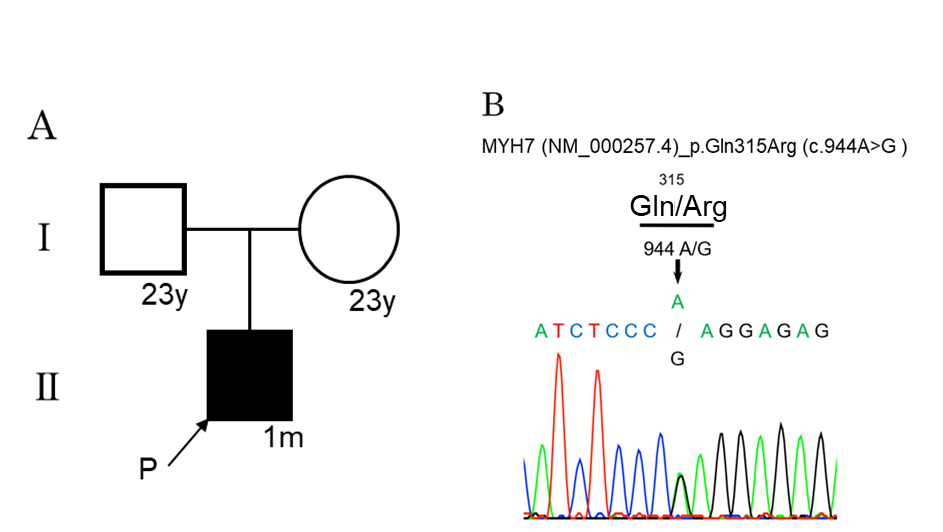

Supplement: S2 Fig — (A) Pedigree of the patient. There is no family history of sudden death or cardiac disease in the parents. (B) DNA sequencing of the patient revealed a single-base missense mutation resulting in the substitution of glutamine (Q) with arginine (R) (black arrow). (DOCX) [file pone.0336131.s002.docx]

**S3 Fig. Comparison of cardiac histology between wild-type and *MYH7* Q315R/+ C57BL/6J strain mice**


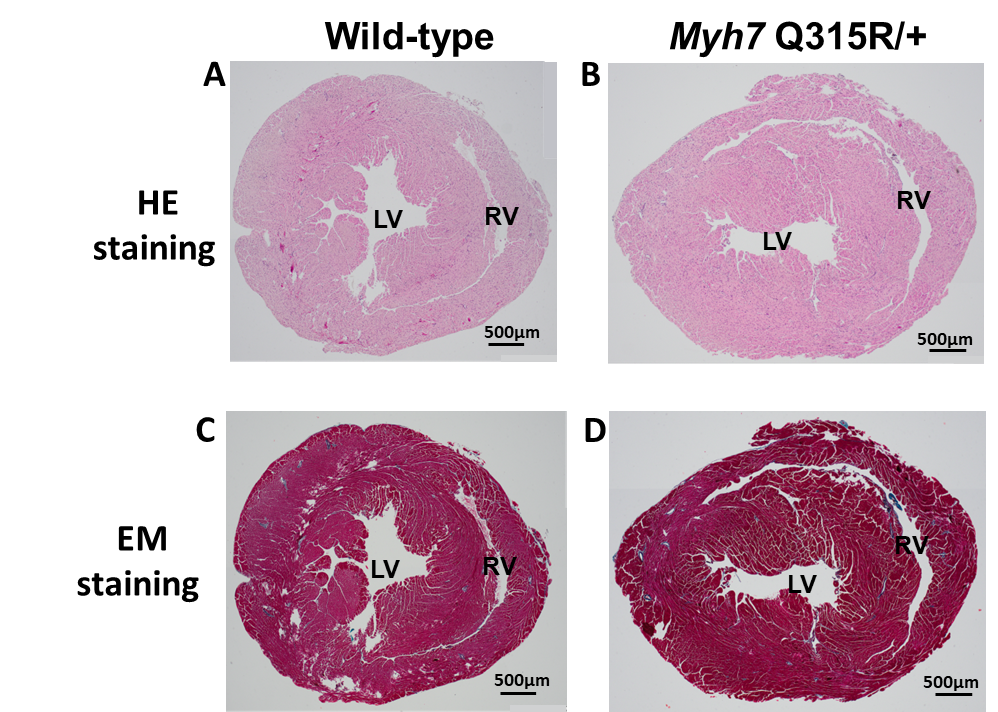

Supplement: S3 Fig — Typical histological hematoxylin-eosin (HE) staining in short-axis images of both groups (A, B). Typical histological Elastica–Masson (EM) staining in short-axis images of both groups (C, D). No significant histological differences were observed between the two groups. RV, right ventricle. LV, left ventricle. (DOCX) [file pone.0336131.s003.docx]

**S4 Fig. Suppression of pentose phosphate pathway metabolites in *MYH7* Q315R mouse hearts**


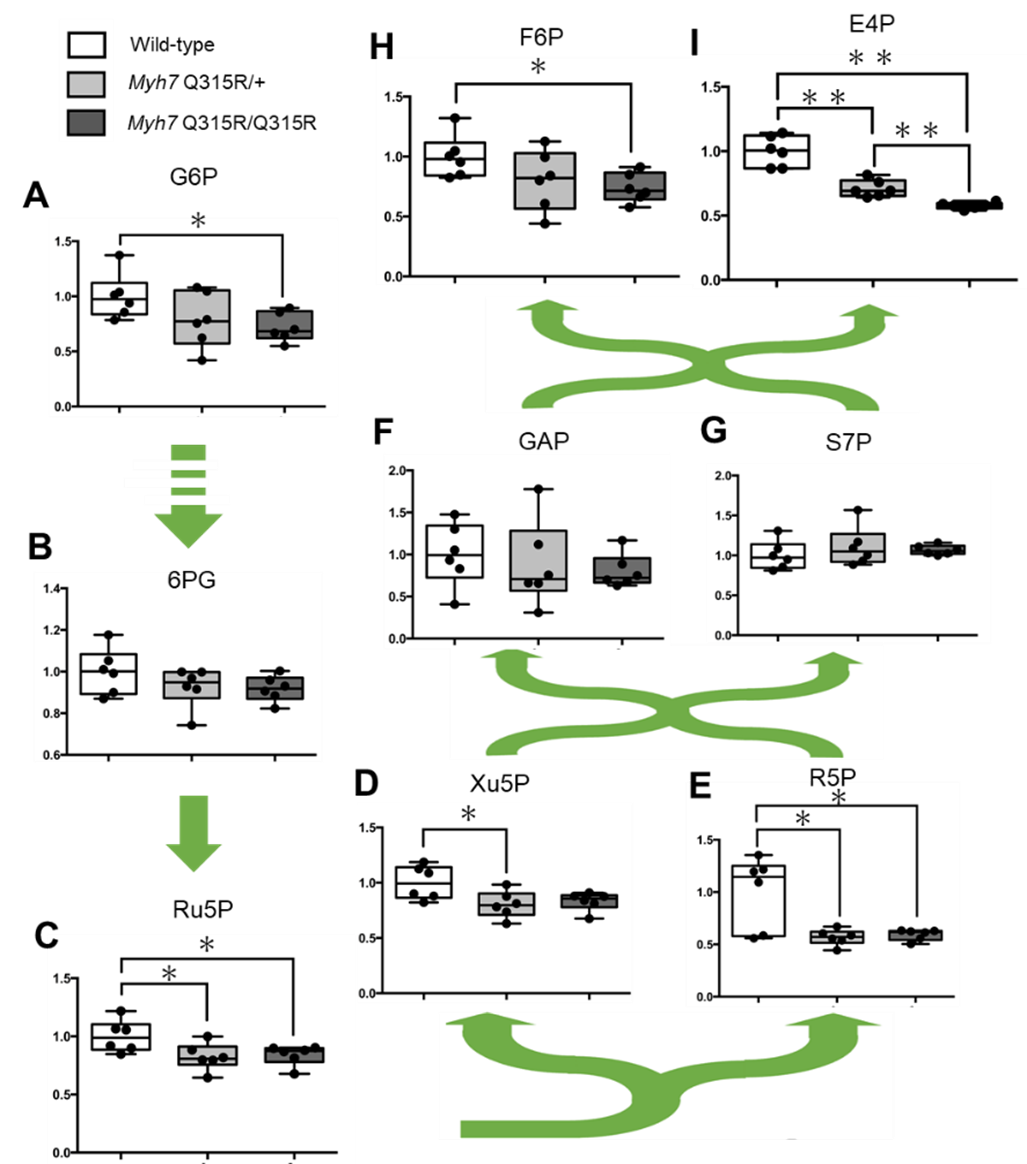

Supplement: S4 Fig — The relative levels of pentose phosphate pathway metabolites in mouse hearts were measured by LC/MS. The scaled intensities of glucose 6-phosphate (A), 6-phosphogluconate (B), ribulose 5-phosphate (C), D-xylulose 5-phosphate (D), ribose 5-phosphate (E), glyceraldehyde 3-phosphate (F), sedoheptulose 7-phosphate (G), fructose 6-phosphate (H), and erythrose 4-phosphate (I) were determined in cell lysates. The main flow of the pentose phosphate pathway is indicated by arrows. G6P, glucose-6-phosphate; 6PG, 6-phosphogluconate; Ru5P, ribulose 5-phosphate; Xu5P, D-xylulose 5-phosphate; R5P, ribose 5-phosphate; GAP, glyceraldehyde 3-phosphate; S7P, sedoheptulose 7-phosphate; F6P, fructose 6-phosphate; E4P, erythrose 4-phosphate. The Box-and-whisker diagrams show the relative fold change compared to the wild-type group. n = 6 per group. * p < 0.05, ** p < 0.01, by unpaired Student’s t-test. (DOCX) [file pone.0336131.s004.docx]

**S5 Fig. Impaired nucleic acid synthesis and salvage pathways in *MYH7* Q315R mice**


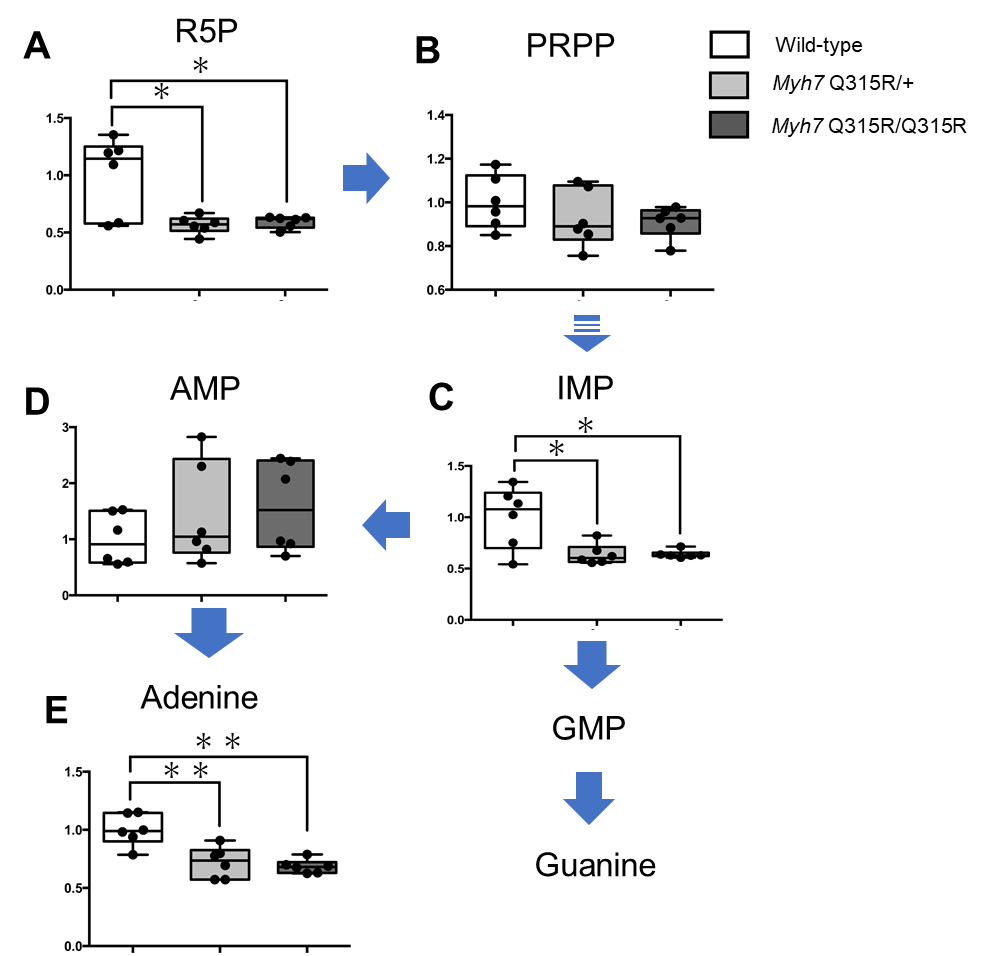

Supplement: S5 Fig — The relative levels of nucleic acid synthesis pathway metabolites and salvage pathway metabolites in mouse hearts were measured by LC/MS. The scaled intensities of ribose 5-phosphate (A), phosphoribosyl diphosphate (B), myo-inositol phosphate (C), adenosine monophosphate (D), and adenine (E) were determined in cell lysates. The main flow of the nucleic acid synthesis pathway is indicated by blue arrows. R5P, ribose 5-phosphate; PRPP, phosphoribosyl diphosphate; IMP, myo-inositol phosphate; AMP, adenosine monophosphate; GMP, guanosine monophosphate. Box-and-whisker diagrams show the relative fold change compared to the wild-type group. n = 6 per group. * p < 0.05, ** p < 0.01, by unpaired Student’s t-test. (DOCX) [file pone.0336131.s005.docx]

**S6 Fig. Altered amino acid profiles in *MYH7* Q315R mice: implications for energy metabolism**

**
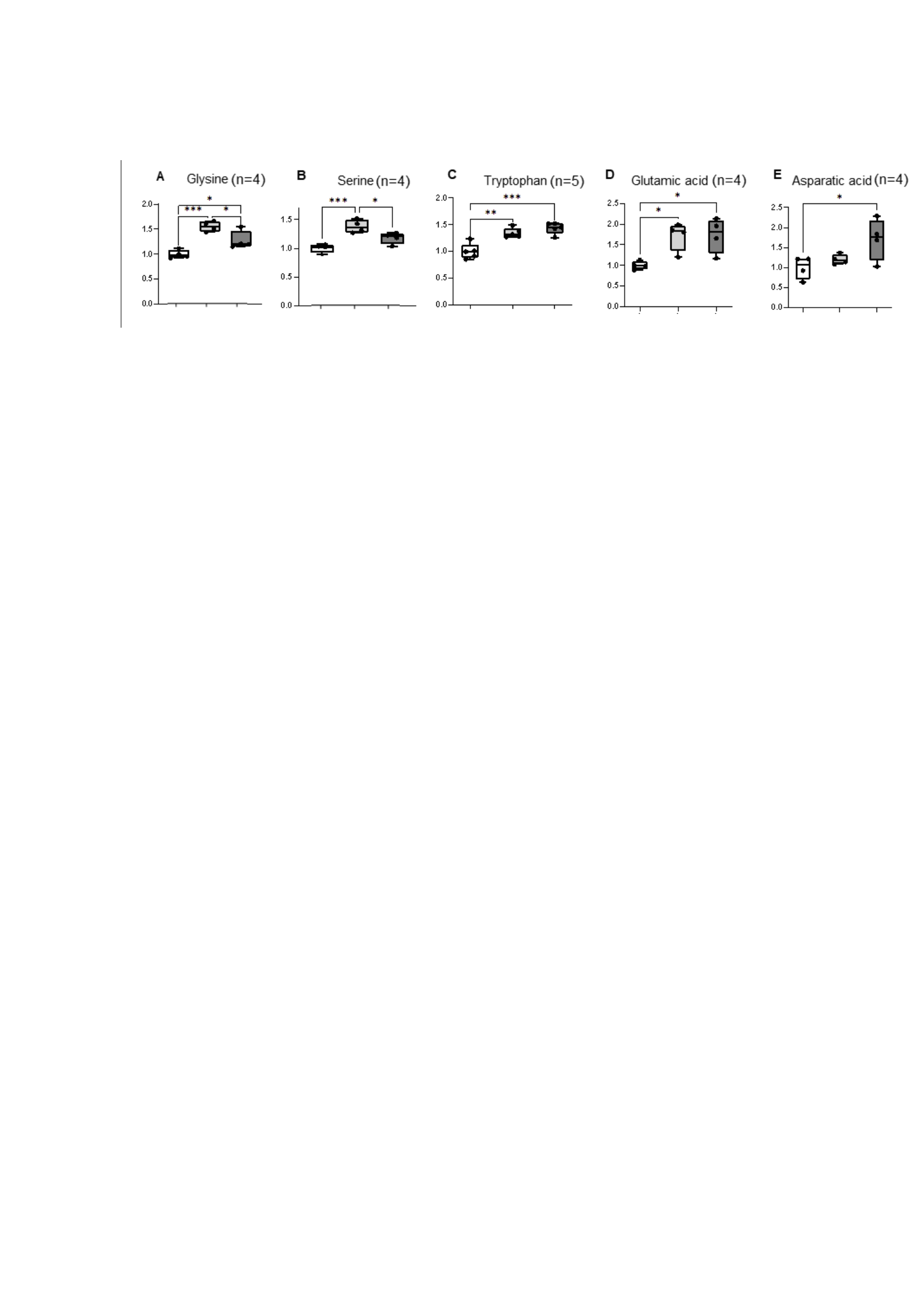
**

Supplement: S6 Fig — Amino acids that differ between MYH7 Q315R variant and wild-type mice include glycine (A), serine (B), tryptophan (C), glutamic acid (D), and aspartic acid (E). The box-and-whisker plots show the relative fold change compared to the wild-type group. The sample size (n) is the same for each group and is indicated in each graph. * p < 0.05, ** p < 0.01, by unpaired Student’s t-test. (DOCX) [file pone.0336131.s006.docx]

**S7 Fig. Metabolic pathways and genes altered in *MYH7* Q315R variant mice**


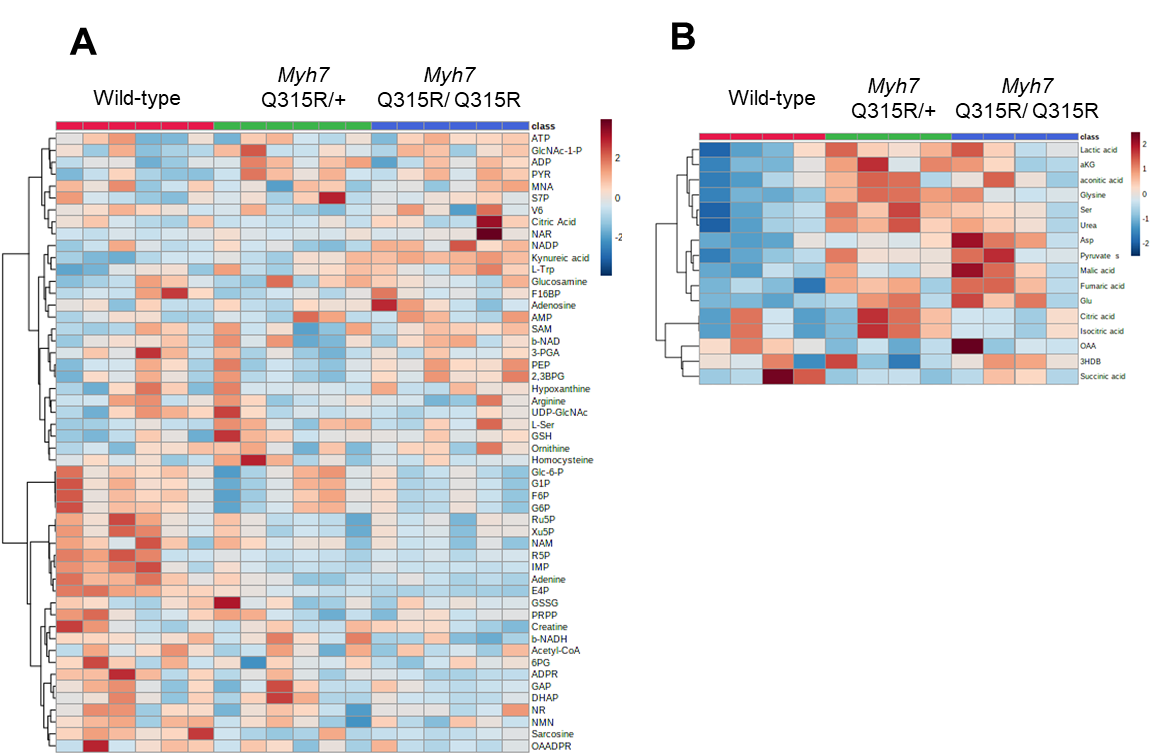

Supplement: S7 Fig — Heat maps clustering the metabolites in each group of mice. A: Heat map clustering the metabolites measured by LC/MS (n = 6 per group). B: Heat map clustering the metabolites measured by GC/MS (n = 4 per group). (DOCX) [file pone.0336131.s007.docx]
