# Supplementary material for: Metabolic remodeling and cardiac dysfunction in left ventricular noncompaction: Insights from the MYH7 Q315R model
Source: PLoS One. 2025 Nov 14;20(11):e0336131. doi: 10.1371/journal.pone.0336131 (PMC12617873; doi:10.1371/journal.pone.0336131)
Supplement: S8 Fig — Sirt2 (A) and Pparγ (B) activate glycolysis and fatty acid oxidation, which were elevated in MYH7 Q315R/ + mice. Pfk (C), Pkm-1 (E), Pdp2 (G), and Bdh1(F) enhance reactions in their respective pathways. Slc27a1 (D) encodes a transport protein that facilitates fatty acid uptake into the cytoplasm. Cpt1a (H) encodes a transferase that transports acetyl-CoA into the mitochondria. Pcg-1α (I) promotes glycolysis, fatty acid oxidation, and mitochondrial function. Cd38 (J) inhibits glycolysis and mitochondrial function. G6P, glucose 6-phosphate; F6P, fructose 6-phosphate; PEP, phosphoenolpyruvic acid; acetyl-CoA, acetyl-coenzyme A; TCA, tricarboxylic acid cycle; FAO, fatty acid oxidation. A–J are graphs showing fold change of each gene in MYH7 Q315R variant mice against the wild type mice. n = 4 for each group. * Fold change >1.2 or <−1.2 and p < 0.05, by unpaired Student’s t-test. (DOCX) [file pone.0336131.s008.docx]

**S8 Fig. Changes in expression levels were observed for genes related to glycolysis, fatty acid oxidation and mitochondrial function in the *MYH7* variant mice**

**
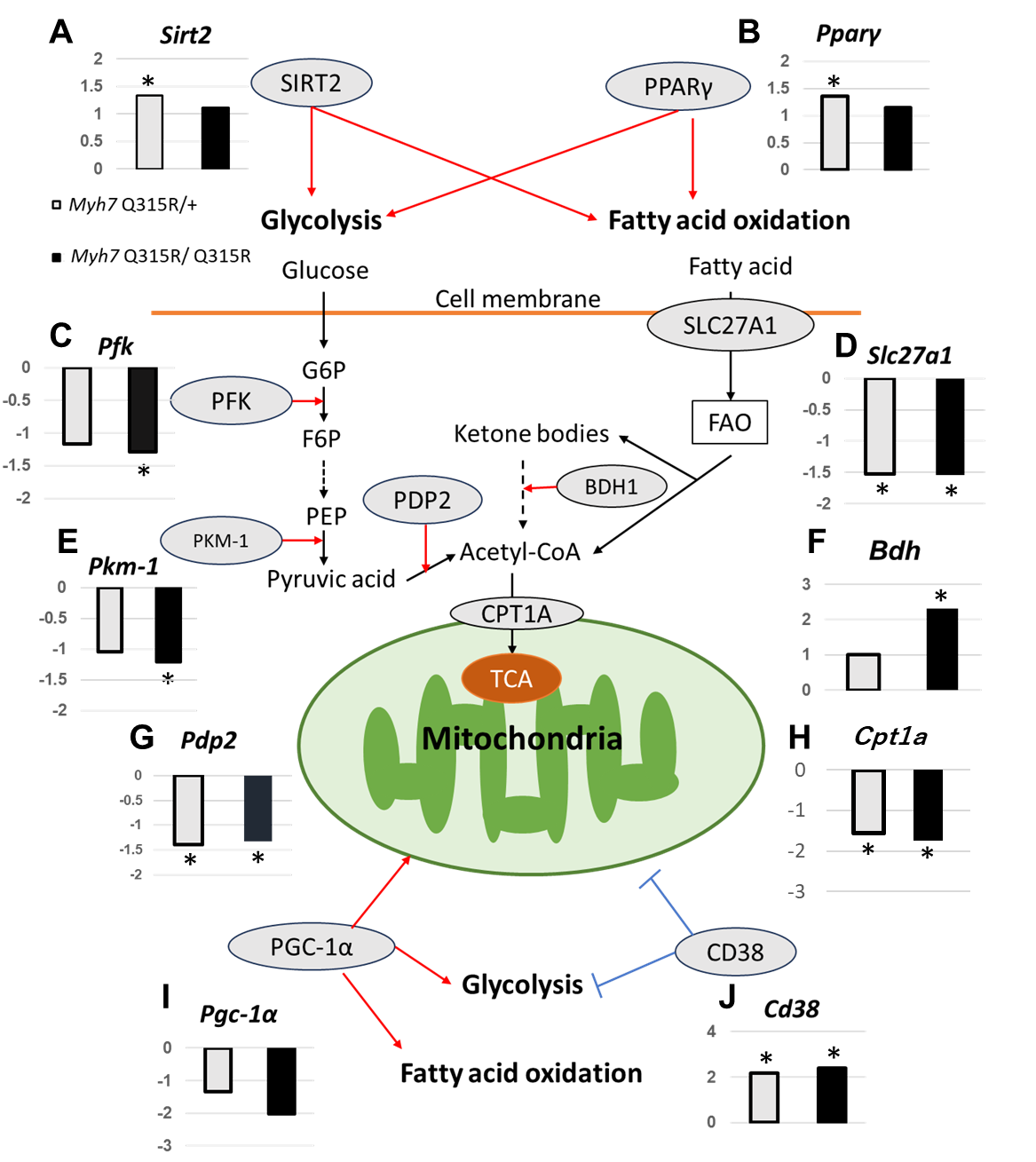
**
