# Supplementary material for: Metabolic remodeling and cardiac dysfunction in left ventricular noncompaction: Insights from the MYH7 Q315R model
Source: PLoS One. 2025 Nov 14;20(11):e0336131. doi: 10.1371/journal.pone.0336131 (PMC12617873; doi:10.1371/journal.pone.0336131)
Supplement: S1 Table — (DOCX) [file pone.0336131.s009.docx]

**S1 Table**. **Primers used in real-time RT-PCR experiments**

| Genes | Sequences (forward primer) | Sequences (reverse primer) |
| --- | --- | --- |
| Mid1 | ctgtgtgaccgatgaccagt | agtttggcttcttgacggga |
| Bpifa1 | gagcctcgttgtcctctgtg | Gcagctgcggatcagtgatt |
| Scgb1a1 | caccaaagcctccaacctct | tcttgcttacacagaggacttgt |
| Postn | gaagggatgaaaggctgccc | tttgtggcaatctggttccc |
| Cd38 | ctctaggaaagcccagatcgg | gtgtcctccagggtgaacat |
| Nppa | gagatgctggcagctaggag | cctgcttcctcagtctgctc |
| Rsad2 | cggaggacacccaggaga | aacctgctcatcgaagctgt |
| Gapdh | ctacccccaatgtgtccgtc | cttcagtgggccctcagatg |
