# Supplementary material for: Metabolic remodeling and cardiac dysfunction in left ventricular noncompaction: Insights from the MYH7 Q315R model
Source: PLoS One. 2025 Nov 14;20(11):e0336131. doi: 10.1371/journal.pone.0336131 (PMC12617873; doi:10.1371/journal.pone.0336131)
Supplement: S2 Table — (DOCX) [file pone.0336131.s010.docx]

**S2 Table. Pathogenicity prediction of the *MYH7* Q315R variant**

| Gene | Accession ID | Protein | cDNA | dbSNP | SIFT | polyphen2 | GVGD | Mutation Taster | FATHMM | CADD | ClinVar |
| --- | --- | --- | --- | --- | --- | --- | --- | --- | --- | --- | --- |
| *MYH7* | NM_000257.4 | p.Gln315Arg | c.944A>G | rs1892890666 | 0 | 0.51 | C35 | Deleterious | −2.44 | 22.1 | Uncertain significance |
