# Supplementary material for: Metabolic remodeling and cardiac dysfunction in left ventricular noncompaction: Insights from the MYH7 Q315R model
Source: PLoS One. 2025 Nov 14;20(11):e0336131. doi: 10.1371/journal.pone.0336131 (PMC12617873; doi:10.1371/journal.pone.0336131)
Supplement: S3 Table — BW, body weight; HR, heart rate; LVEDD, left ventricular end-diastolic diameter; LVEDs, left ventricular end-systolic diameter; AWD, left ventricular anterior wall thickness at end diastole; PWD, left ventricular posterior wall thickness at end diastole; FS, fractional shortening; E, trans-mitral early wave; A, trans-mitral atrial wave; and HW, heart weight. Values significantly different from wild-type are indicated. (DOCX) [file pone.0336131.s011.docx]

**S3 Table. Baseline echocardiographic parameters in wild-type and *MYH7* Q315R/+ C57BL/6J strain mice**

|  | Wild-type | *MYH7* Q315R /+ | *p*-value |
| --- | --- | --- | --- |
| BW (g) | 20.5 ± 2.85 | 21.3 ± 3.15 | 0.6417 |
| HR (min) | 447.0 ± 28.1 | 460.5 ± 29.5 | 0.7115 |
| LVDd (mm) | 3.91 ± 0.12 | 3.98 ± 0.12 | 0.6910 |
| LVDs (mm) | 3.09 ± 0.21 | 2.90 ± 0.24 | 0.1700 |
| AWD (mm) | 0.77 ± 0.15 | 0.9 ± 0.06 | 0.1416 |
| PWD (mm) | 0.77 ± 0.05 | 0.85 ± 0.05 | 0.3047 |
| FS (%) | 21.2 ± 1.9 | 27.0 ± 5.77 | 0.0287 |
| E (cm/s) | 81.2 ± 7.14 | 85.1 ± 6.61 | 0.6997 |
| A (cm/s) | 34.0 ± 8.65 | 59.7 ± 32.94 | 0.0711 |
| E/A (cm/s) | 2.66 ± 0.24 | 1.49 ± 0.25 | 0.0060 |
| HW (mg) | 103.4 ± 2.94 | 104.5 ± 4.14 | 0.8090 |
| HW/BW | 5.09 ± 0.46 | 4.99 ± 0.60 | 0.7280 |
