# Supplementary material for: Metabolic remodeling and cardiac dysfunction in left ventricular noncompaction: Insights from the MYH7 Q315R model
Source: PLoS One. 2025 Nov 14;20(11):e0336131. doi: 10.1371/journal.pone.0336131 (PMC12617873; doi:10.1371/journal.pone.0336131)
Supplement: S4 Table — BW, body weight; HR, heart rate; LVEDD, left ventricular end-diastolic diameter; LVEDs, left ventricular end-systolic diameter; AWD, left ventricular anterior wall thickness at end diastole; PWD, left ventricular posterior wall thickness at end diastole; FS, fractional shortening; E, trans-mitral early wave; A, trans-mitral atrial wave; and HW, heart weight. Values significantly different from wild-type and between MYH7 Q315R/+ and MYH7 Q315R/Q315R mice are indicated. (DOCX) [file pone.0336131.s012.docx]

**S4 Table. Echocardiographic data and heart weight of young adult wild-type, *MYH7* Q315R/+, and *MYH7* Q315R/Q315R mice with isoproterenol loading**

|  | Wild-type | *MYH7* Q315R /+ | *MYH7* Q315R/Q315R | *p*-value |
| --- | --- | --- | --- | --- |
| BW (g) | 23.1 ± 2.5 | 26.0 ± 2.2 | 23.7 ± 2.6 | 0.1322 |
| HR (/min) | 400.4 ± 18.1 | 407.2 ± 29.6 | 411.6 ± 28.7 | 0.7567 |
| LVDd (mm) | 4.1 ± 0.26 | 4.5 ± 0.32 | 4.3 ± 0.21 | 0.0730 |
| LVDs (mm) | 2.9 ± 0.36 | 3.5 ± 0.40^†^ | 3.4 ± 0.2^†^ | 0.0213 |
| AWD (mm) | 0.63 ± 0.10 | 0.63 ± 0.10 | 0.52 ± 0.08 | 0.0788 |
| PWD (mm) | 0.65 ± 0.11 | 0.67 ± 0.05 | 0.55 ± 0.08 | 0.0578 |
| FS (%) | 30.1 ± 4.5 | 23.8 ± 4.3^†^ | 20.9 ± 4.3 | 0.0040 |
| E (cm/s) | 79.6 ± 18.0 | 65.4 ± 4.8 | 63.6 ± 5.2 | 0.1251 |
| A (cm/s) | 37.5 ± 9.6 | 46.3 ± 10.3 | 45.5 ± 7.5 | 0.3265 |
| E/A (cm/s) | 2.19 ± 0.58 | 1.45 ± 0.23 | 1.45 ± 0.37 | 0.0214 |
| HW (mg) | 116.7 ± 11.1 | 141.7 ± 10.1 | 122.2 ± 12.0 | 0.0099 |
| HW/BW | 5.03 ± 0.26 | 5.51 ± 0.24 | 5.18 ± 0.33 | 0.0298 |
