# Supplementary material for: Metabolic remodeling and cardiac dysfunction in left ventricular noncompaction: Insights from the MYH7 Q315R model
Source: PLoS One. 2025 Nov 14;20(11):e0336131. doi: 10.1371/journal.pone.0336131 (PMC12617873; doi:10.1371/journal.pone.0336131)
Supplement: S5 Table — C layer, compacted layer; NC layer, noncompacted layer; NC/C ratio, ratio of the thickness of the NC layer divided by the thickness of the C layer. Values significantly different from wild-type are indicated. (DOCX) [file pone.0336131.s013.docx]

**S5 Table. Segmental variations in NC/C ratios across the left ventricle in *MYH7* Q315R mice**

| Segmentations | Measured items | Wild**-**type | *MYH7* Q315R /+ | *MYH7* Q315R/Q315R | *p*-value |
| --- | --- | --- | --- | --- | --- |
| Anterior | Wall thickness | 0.56 ± 0.09 | 0.50 ± 0.15 | 0.61 ± 0.08 | 0.3269 |
|  | C layer | 0.42 ± 0.05 | 0.38 ± 0.15 | 0.43 ± 0.04 | 0.6743 |
|  | NC layer | 0.14 ± 0.07 | 0.12 ± 0.05 | 0.19 ± 0.08 | 0.3602 |
|  | NC/C ratio | 0.33 ± 0.16 | 0.41 ± 0.25 | 0.45 ± 0.20 | 0.6849 |
| Anterolateral | Wall thickness | 0.67 ± 0.04 | 0.64 ± 0.22 | 0.76 ± 0.07 | 0.3585 |
|  | C layer | 0.53 ± 0.04 | 0.43 ± 0.16 | 0.50 ± 0.03 | 0.2796 |
|  | NC layer | 0.14 ± 0.02 | 0.20 ± 0.07 | 0.26 ± 0.06^†^ | 0.0153 |
|  | NC/C ratio | 0.26 ± 0.06 | 0.47 ± 0.04^†^ | 0.51 ± 0.14^††^ | 0.0020 |
| Posterolateral | Wall thickness | 0.71 ± 0.09 | 0.63 ± 0.15 | 0.74 ± 0.12 | 0.3730 |
|  | C layer | 0.54 ± 0.05 | 0.44 ± 0.09 | 0.48 ± 0.06 | 0.1049 |
|  | NC layer | 0.17 ± 0.05 | 0.19 ± 0.07 | 0.26 ± 0.09 | 0.1529 |
|  | NC/C ratio | 0.31 ± 0.07 | 0.43 ± 0.12 | 0.54 ± 0.18 ^†^ | 0.0456 |
| Posterior | Wall thickness | 0.63 ± 0.05 | 0.55 ± 0.13 | 0.57 ± 0.04 | 0.2572 |
|  | C layer | 0.48 ± 0.08 | 0.43 ± 0.10 | 0.46 ± 0.07 | 0.6275 |
|  | NC layer | 0.15 ± 0.06 | 0.12 ± 0.09 | 0.11 ± 0.05 | 0.5699 |
|  | NC/C ratio | 0.34 ± 0.17 | 0.30 ± 0.23 | 0.25 ± 0.15 | 0.7661 |
| Posteroseptal | Wall thickness | 0.59 ± 0.07 | 0.53 ± 0.16 | 0.64 ± 0.07 | 0.3304 |
|  | C layer | 0.51 ± 0.08 | 0.46 ± 0.14 | 0.57 ± 0.08 | 0.3363 |
|  | NC layer | 0.08 ± 0.03 | 0.07 ± 0.03 | 0.08 ± 0.02 | 0.9734 |
|  | NC/C ratio | 0.16 ± 0.08 | 0.16 ± 0.04 | 0.14 ± 0.06 | 0.8724 |
| Anteroseptal | Wall thickness | 0.58 ± 0.08 | 0.46 ± 0.11 | 0.63 ± 0.04 | 0.0379 |
|  | C layer | 0.49 ± 0.09 | 0.38 ± 0.10 | 0.53 ± 0.03 | 0.0414 |
|  | NC layer | 0.09 ± 0.03 | 0.08 ± 0.05 | 0.10 ± 0.03 | 0.7109 |
|  | NC/C ratio | 0.20 ± 0.08 | 0.20 ± 0.12 | 0.18 ± 0.05 | 0.8956 |
