# Supplementary material for: Metabolic remodeling and cardiac dysfunction in left ventricular noncompaction: Insights from the MYH7 Q315R model
Source: PLoS One. 2025 Nov 14;20(11):e0336131. doi: 10.1371/journal.pone.0336131 (PMC12617873; doi:10.1371/journal.pone.0336131)
Supplement: S6 Table — The fibrotic tissue area (%) is calculated by dividing fibrotic tissue area(mm2) by the total area (mm2) for each group. No significant differences were observed between the three group. (DOCX) [file pone.0336131.s014.docx]

**S6 Table. Quantitative analysis of myocardial fibrosis in *MYH7* Q315R mouse groups with isoproterenol loading**

|  | Wild**-**type | *MYH7* Q315R /+ | *MYH7* Q315R/Q315R | *p*-value |
| --- | --- | --- | --- | --- |
| Total area (mm^2^) | 1.83 ± 0.30 | 1.87 ± 0.14 | 1.87 ± 0.18 | 0.9609 |
| Fibrosis area (mm^2^) | 0.17 ± 1.91 | 0.15 ± 0.04 | 0.15 ± 0.03 | 0.7786 |
| Area (%) | 8.94 ± 1.91 | 7.87 ± 1.02 | 7.77 ± 1.02 | 0.5875 |
