# Supplementary material for: Metabolic remodeling and cardiac dysfunction in left ventricular noncompaction: Insights from the MYH7 Q315R model
Source: PLoS One. 2025 Nov 14;20(11):e0336131. doi: 10.1371/journal.pone.0336131 (PMC12617873; doi:10.1371/journal.pone.0336131)
Supplement: S7 Table — The table above shows the results for the MYH7 Q315R/+ group. The table below shows the results for the MYH7 Q315R/ Q315R group. (DOCX) [file pone.0336131.s015.docx]

| Source | Term name | Term id | Adjusted *P-* value | Negative log10 of  Adjusted*P-* value | Term size | Query size | Intersection size | Effective domain size | Intersections |
| --- | --- | --- | --- | --- | --- | --- | --- | --- | --- |
| GO:BP | Response to cytokine | GO:0034097 | 0.004538 | 2.343101 | 935 | 22 | 8 | 21118 | SCGB1A1,IFIT3B,GBP11,CCL9,CCL6,POSTN,CD38,GBP6 |
| GO:BP | Cellular response to interferon-gamma | GO:0071346 | 0.011176 | 1.951723 | 119 | 22 | 4 | 21118 | GBP11,CCL9,CCL6,GBP6 |
| GO:BP | Immune response | GO:0006955 | 0.011194 | 1.95102 | 1857 | 22 | 10 | 21118 | BPIFA1,IFI44,TLR13,IFIT3B,GBP11,CCL9,CCL6,CD38,GBP6,NPPA |
| GO:BP | Response to external stimulus | GO:0009605 | 0.013385 | 1.87337 | 2912 | 22 | 12 | 21118 | BPIFA1,UCP3,SCGB1A1,IFI44,TLR13,IFIT3B,GBP11,CCL9,CCL6,POSTN,GBP6,NPPA |

**S7 Table. Functional enrichment analysis of differentially expressed genes in *MYH7* Q315R mice**

| Source | Term name | Term id | Adjusted *p-* value | Negative log10 of  Adjusted*P-* value | Term size | Query size | Intersection size | Effective domain size | Intersections |
| --- | --- | --- | --- | --- | --- | --- | --- | --- | --- |
| GO:BP | Defense response | GO:0006952 | 3.89E-05 | 4.409551 | 1705 | 44 | 17 | 21118 | BPIFA1,SCGB1A1,DDIT4,CTSC,S100A8,APOBEC1,TLR13,CLEC7A,C1QB,CASP1,CYSLTR1,PLA2G7,GBP2,C3AR1,LY96,RSAD2,GBP6 |
| GO:BP | Response to stress | GO:0006950 | 0.000108 | 3.966735 | 3817 | 44 | 24 | 21118 | MID1,SIK1,BPIFA1,SCGB1A1,TRP53INP1,DDIT4,CTSC,S100A8,APOBEC1,POSTN,TLR13,CLEC7A,C1QB,CASP1,CD38,PLK2,CYSLTR1,PLA2G7,GBP2,C3AR1,FMO1,LY96,RSAD2,GBP6 |
| GO:BP | Response to external stimulus | GO:0009605 | 0.000125 | 3.904427 | 2912 | 44 | 21 | 21118 | SIK1,BPIFA1,SCGB1A1,DDIT4,CTSC,S100A8,APOBEC1,POSTN,TLR13,CLEC7A,C1QB,CASP1,CYSLTR1,HPGD,PLA2G7,GBP2,C3AR1,FMO1,LY96,RSAD2,GBP6 |
| GO:BP | Immune response | GO:0006955 | 0.000137 | 3.862156 | 1857 | 44 | 17 | 21118 | BPIFA1,DDIT4,CTSC,S100A8,APOBEC1,TLR13,CLEC7A,C1QB,CASP1,CD38,CYSLTR1,GBP2,C3AR1,LY96,PTGDS,RSAD2,GBP6 |
