# Supplementary material for: Metabolic remodeling and cardiac dysfunction in left ventricular noncompaction: Insights from the MYH7 Q315R model
Source: PLoS One. 2025 Nov 14;20(11):e0336131. doi: 10.1371/journal.pone.0336131 (PMC12617873; doi:10.1371/journal.pone.0336131)
Supplement: S8 Table — Fold change and p-value were compared with wild-type mice. n = 4 per group. p-value was determined by unpaired Student’s t-test. (DOCX) [file pone.0336131.s016.docx]

**S8 Table. Alterations in *MYH7* Q315R variant mice genes associated with metabolism, as revealed by microarray analysis**

|  | *MYH7* Q315R/+ mice | | *MYH7* Q315R/Q315R mice | |
| --- | --- | --- | --- | --- |
|  | Fold change | *p*-value | Fold change | *p*-value |
| Pdp2 | -1.39 | 0.0232 | -1.33 | 0.0317 |
| Pfk | -1.17 | 0.09 | -1.29 | 0.0304 |
| Pkm1 | -1.04 | 0.5925 | -1.21 | 0.0417 |
| Cpt1a | -1.55 | 0.0836 | -1.74 | 0.0015 |
| Slc27a1 | -1.53 | 0.0062 | -1.54 | 0.006 |
| Bdh | 1 | 0.9622 | 2.31 | 0.0367 |
| Cd38 | 2.16 | 0.0034 | 2.4 | 0.0042 |
| Sirt2 | 1.34 | 0.0417 | 1.12 | 0.3946 |
| Pparγ | 1.36 | 0.0262 | 1.15 | 0.3865 |
